# Novel architecture for gated recurrent unit autoencoder trained on time series from electronic health records enables detection of ICU patient subgroups

**DOI:** 10.1038/s41598-023-30986-1

**Published:** 2023-03-11

**Authors:** Kilian Merkelbach, Steffen Schaper, Christian Diedrich, Sebastian Johannes Fritsch, Andreas Schuppert

**Affiliations:** 1 JRC-COMBINE, RWTH Aachen University, MTZ, Pauwelsstrasse 19, Level 3, 52074 Aachen, Germany; 2 Pharmacometrics / Modeling and Simulation, Bayer AG - Pharmaceuticals, Leverkusen, Germany; 3 Department of Intensive Care Medicine, University Hospital RWTH Aachen, Pauwelsstrasse 30, 52074 Aachen, Germany; 4 Juelich Supercomputing Centre, Forschungszentrum Juelich, Wilhelm-Johnen-Straße, 52428 Juelich, Germany

**Keywords:** Computer science, Medical research

## Abstract

Electronic health records (EHRs) are used in hospitals to store diagnoses, clinician notes, examinations, lab results, and interventions for each patient. Grouping patients into distinct subsets, for example, via clustering, may enable the discovery of unknown disease patterns or comorbidities, which could eventually lead to better treatment through personalized medicine. Patient data derived from EHRs is heterogeneous and temporally irregular. Therefore, traditional machine learning methods like PCA are ill-suited for analysis of EHR-derived patient data. We propose to address these issues with a new methodology based on training a gated recurrent unit (GRU) autoencoder directly on health record data. Our method learns a low-dimensional feature space by training on patient data time series, where the time of each data point is expressed explicitly. We use positional encodings for time, allowing our model to better handle the temporal irregularity of the data. We apply our method to data from the Medical Information Mart for Intensive Care (MIMIC-III). Using our data-derived feature space, we can cluster patients into groups representing major classes of disease patterns. Additionally, we show that our feature space exhibits a rich substructure at multiple scales.

## Introduction

Hospitals and other healthcare providers collect and store medical data on their patients in electronic health records (EHR) to document the course of their diseases and treatment. These measurements may include, among others, vital signs, the doses and types of medication administered, and laboratory or diagnostic parameters. As opposed to data assessed in a clinical study, each patient’s EHR provides different attributes of data since caregivers assess the necessary examinations for every new patient individually.

These complex time series contain a wealth of latent information. It would be beneficial for clinicians and researchers to be able to represent the totality of a patient’s data in a low-dimensional form. This would enable comparisons and groupings of individual patients and serve as a stepping stone for other methods, such as outcome prediction or monitoring.

The nature of such data, however, brings with it the following challenges, which traditional machine learning algorithms are ill-suited to handle^1^:

1. *Irregularity:* Patient data is not captured on a time-regular grid but assessed when deemed necessary by caregivers in charge of the patient.
2. *Sparsity:* Only a small number of data attributes are available for all patients since the examinations, diagnostics, and treatments are coordinated by caregivers according to the individual patient’s needs. The overwhelming majority of data attributes only have information for a minority of the patients. Additionally, the fact that a data attribute is missing may be informative.
3. *High dimensionality:* There exist many different attributes of data in use in critical care.

In this work, we address these challenges with an unsupervised deep learning method that learns a compact feature space for each admission into the intensive care unit (ICU). The proposed method can be used on the raw patient data without temporal interpolation and without the assumption that the same data attributes are available for all patients. This flexibility allows the method to form a consistent view of the patient’s condition using data from vastly different time scales. Importantly, our method learns to compress time series of clinical data into a flat, time-independent representation, from which it can reconstruct the original time series’ dynamics again. Note that we reconstruct all of the time series belonging to an ICU admission using a single representation corresponding to the admission. Thus, many time series are reconstructed from a single point in our learned feature space. This dimensionality reduction is learned directly from the patient data. Importantly, this compression and reconstruction are lossy by design, which forces the neural network to distill regularities and patterns of the raw data into the learned feature space. We show that we can identify clinically meaningful groups by clustering the patients based on our trained feature space. The source code for our method is freely available (at https://github.com/JRC-COMBINE/ehr-time-series-gru-autoencoder).

The paper is structured as follows: Following the introduction, we present reconstruction and clustering results. Then we discuss the results. Finally, the methods section provides an overview of our method, the data we utilized, and the details of the machine learning model we employed. In the following, we first discuss related work.

Due to the high density of data points which hospitals store during medical treatment, the field of Intensive Care Medicine offers excellent preconditions for developing and applying machine learning models. Thus, many works employing machine learning in critical care have been published in recent years^2–4^. However, most of these works use labeled data, for example, ICD-9 diagnoses, and thus fall under the umbrella of supervised learning approaches. They usually predict a clinical event of some kind—mortality, specific diagnoses, or the likelihood of re-admissions into the hospital^5–10^. However, since these studies follow the paradigm of supervised learning, they are limited in what they can find. In contrast, unsupervised learning methods forego the use of such labels and by this means also avoid problems with incorrect or untimely diagnoses, negligent documentation, and heterogeneous terminology^11^. A strength of unsupervised learning lies in the ability to gain insight into complex, multi-dimensional data sets which might be beyond the capabilities of human perception. Despite the good availability of such data sets, there is only a small number of publications applying unsupervised methods on Intensive Care data sets compared to supervised learning models. Vranas et al.^12^ applied a clustering analysis on a heterogeneous Intensive Care population and found six clinically recognizable clusters. Other researchers used electronic health record data, including diagnoses, for clustering analysis to identify latent disease clusters or phenotypes, partially limiting their approach to patients with distinct diagnoses like sepsis or COVID-19^13–15^. Hyun et al., however, could show that clustering was possible using exclusively laboratory test data without knowledge about the diagnoses^16^. However, we note that dynamic data may contain the relevant information in their temporal course, making it impossible to choose a particular time point to generate a “snapshot” as input for a conventional machine learning model. An autoencoder can represent a time series of dynamic data in its feature space, including all relevant information from the whole length of stay. Beaulieu-Jones et al. used information on care events from the MIMIC-III data set as input data for an autoencoder and were able to find meaningful clusterings of mortality^17^. Deep autoencoders have been applied to the problem of dimensionality reduction in many domains of research, including image processing, where they can be used to generate new images^18–20^ and engineering^21,22^.

Time series in the medical domain are often very sparse and irregular in the temporal dimension. This is because the intervals between observations (e.g., blood count) depend on the patient’s clinical condition and the doctors’ assessment of this situation. While some methods quantize the temporal dimension onto a regular grid^23–25^, recently, there has been a growing trend of utilizing raw time series^9,26^. This has the benefit of preserving the information contained in the missingness and sampling times of variables. Additionally, resampling to a regular temporal grid requires striking a compromise between a fine grid, which will end up very sparse, and a coarse grid, which will group many distinct observations.

## Results

We evaluate the reconstruction fidelity by performing reconstruction of the original time series the same way as during training. Since our goal is a dimensionality reduction, the reconstruction is expected to deviate from the original time series. Within normalized space, we calculate the mean squared error (MSE) between each ground truth time series and its reconstruction. We take the median over all time series and arrive at a final reconstruction error of 0.25. If mean absolute percentage error (MAPE) is used instead of MSE, the reconstruction error is $9.28\%$.

**Figure 1:**
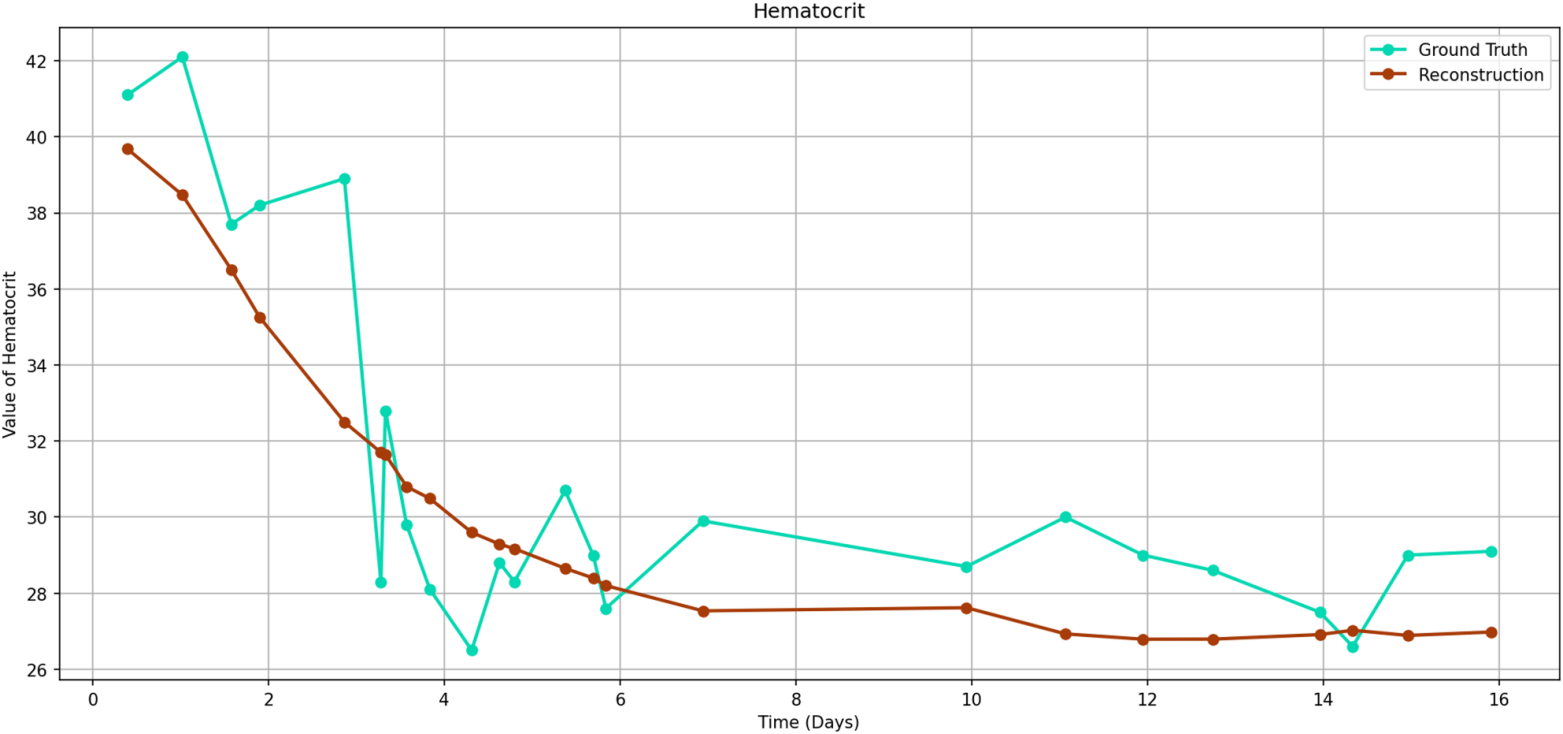
Reconstruction example for *Hematocrit*. The mean squared error (MSE) of this reconstruction is 0.23, which is representative of the model, which has a median reconstruction MSE of 0.25. Note how high-frequency movements (e.g., downward spike at around three days) are not reconstructed, but large-scale movements are. This indicates that the model has learned to grasp the “trend” of the time series, making the feature space used for reconstruction more meaningful.

We show an exemplary reconstruction plot in Fig. 1 and additional plots in the supplementary materials.

**Figure 2:**
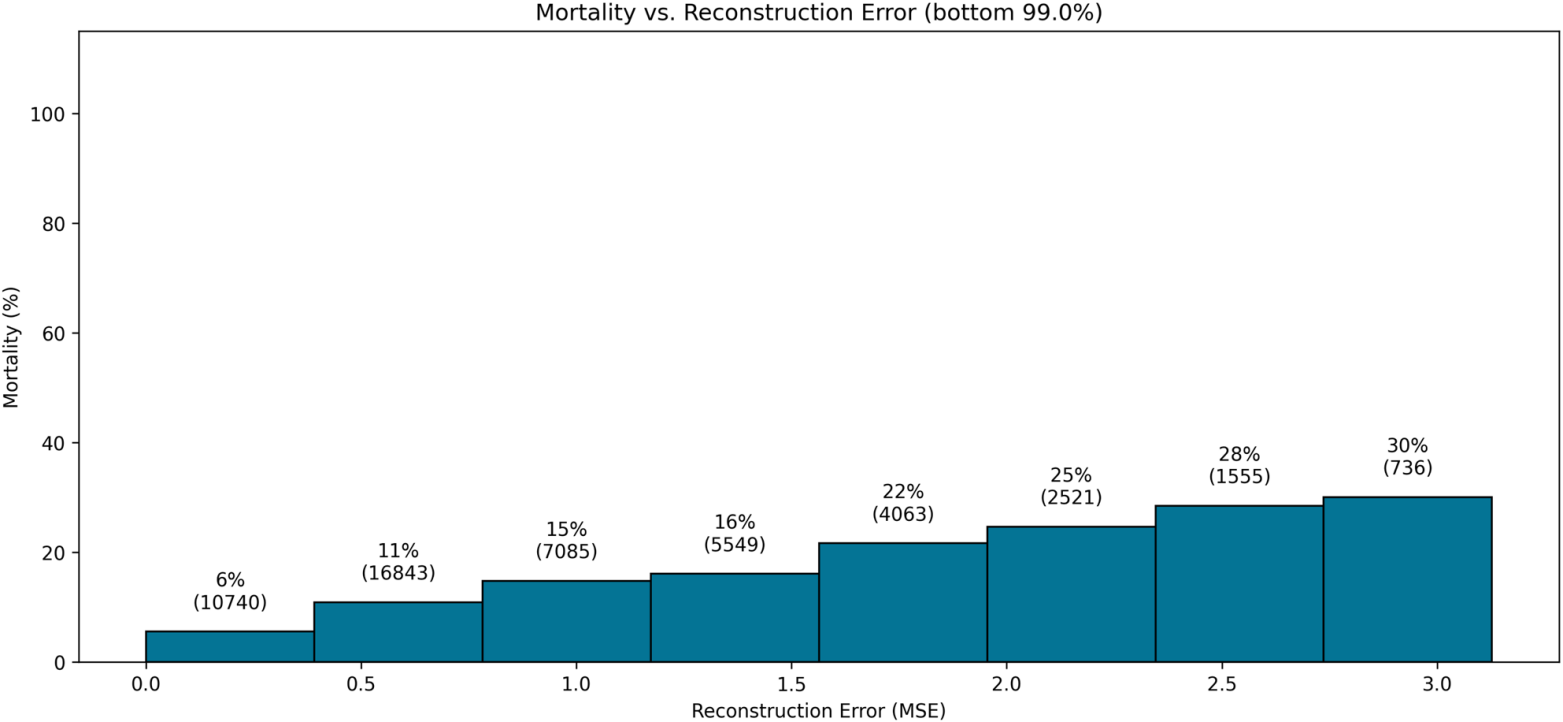
Relationship between reconstruction error and mortality. For every admission, we assess the mean squared reconstruction error over all time steps and the 28-day mortality. We show admissions within different ranges of reconstruction error as bars, with the fraction of admissions with mortality and the total count of admissions displayed above each bar. On average, admissions with higher reconstruction error have higher mortality: Between the admissions with low reconstruction error and those with high reconstruction error, there is a five-fold increase in mortality. For this plot, we only include the bottom $99\%$ of admissions (concerning the reconstruction error).

Additionally, we demonstrate that admissions with higher reconstruction error also have higher than average mortality (see Fig. 2).

Since the model can reconstruct a close approximation of the original time series from the learned feature space, it must contain the medical information necessary for this process. Following this line of thought, we examine the results of clustering on the feature space. The patient population in MIMIC is heterogeneous and multifaceted, so our approach is to identify multiple clusterings at different *k*. In particular, we present two clusterings: a coarse clustering with $k=2$ and a finer clustering with $k=6$. Coarse clustering results are shown in Table 1, while the primary clustering results are shown in Table 2.

Note that the distribution of length of stay in the ICU over the clusters can also be of interest since it can indicate diseases where therapy takes longer. For the patient’s age and the number of days spent in the ICU, we provide the median in addition to the first and third quartile ($Q_1$ and $Q_3$). Mortality is defined as the 28-day mortality, according to which patients are considered deceased if they expire within 28 days of admission. In this case, the mortality registration is not limited to the hospital but is also recorded if the patient dies outside the hospital.

**Table 1:** Coarse clustering results.

Coarse cluster name	Descriptive statistics	Prevalent diagnoses	Enriched diagnoses	Prevalent procedures	Enriched procedures
Population	N = 49599 43.9% female Age: 66 years (53, 78) ICU stay - all: 2 days (1, 4) - survivors: 2 days (1, 4) - deceased: 3 days (1, 6) 28-day mortality: 13.6%	401.9 - Unspecified essential hypertension (41.3%), 414.0 Coronary atherosclerosis (27.9%), 427.3 Atrial fibrillation and flutter (26.6%), 428.0 - Congestive heart failure, unspecified (26.2%), 250.0 Diabetes mellitus without mention of complication or manifestation classifiable to 250.1–250.9 (19.7%), 518.8 Other disease of lung (19.5%), 584.9 - Acute kidney failure, unspecified (18.2%), 272.4 - Other and unspecified hyperlipidemia (17.4%), V58.6 Long-term (current) drug use (14.1%), 530.8 Other specified disorders of esophagus (13.2%), 599.0 - Urinary tract infection, site not specified (13.1%), V45.8 Other postprocedural status (12.3%), 403.9 Unspecified hypertensive kidney disease (12.1%), 272.0 - Pure hypercholesterolemia (11.8%), 995.9 Systemic inflammatory response syndrome (SIRS) (10.8%)	–	38.93 - Venous catheterization, not elsewhere classified (25.1%), 96.04 - Insertion of endotracheal tube (17.0%), 96.71 - Continuous invasive mechanical ventilation for less than 96 consecutive hours (15.6%), 96.6 - Enteral infusion of concentrated nutritional substances (14.9%), 99.04 - Transfusion of packed cells (13.8%), 39.61 - Extracorporeal circulation auxiliary to open heart surgery (13.6%), 96.72 - Continuous invasive mechanical ventilation for 96 consecutive hours or more (11.2%), 88.56 - Coronary arteriography using two catheters (10.2%), 36.15 - Single internal mammary-coronary artery bypass (8.8%), 38.91 - Arterial catheterization (8.7%), 88.72 - Diagnostic ultrasound of heart (6.6%), 37.22 - Left heart cardiac catheterization (6.4%), 39.95 - Hemodialysis (6.4%), 99.15 - Parenteral infusion of concentrated nutritional substances (5.9%), 33.24 - Closed [endoscopic] biopsy of bronchus (5.3%)	–
Cluster 1	N = 19147 78.1% female Age: 69 years (55, 81) ICU stay - all: 3 days (1, 6) - survivors: 3 days (1, 6) - deceased: 3 days (1, 7) 28-day mortality: 23.5%	$\sim$ 401.9 - Unspecified essential hypertension (40.3%, -3.9%), + 518.8 Other disease of lung (29.2%, +118.1%), + 428.0 - Congestive heart failure, unspecified (28.5%, +15.1%), $\sim$ 427.3 Atrial fibrillation and flutter (27.2%, +3.9%), + 584.9 - Acute kidney failure, unspecified (21.1%, +28.4%), − 414.0 Coronary atherosclerosis (20.3%, -38.0%), $\sim$ 250.0 Diabetes mellitus without mention of complication or manifestation classifiable to 250.1-250.9 (19.8%, +1.3%), + 599.0 - Urinary tract infection, site not specified (18.2%, +84.1%), $\sim$ 272.4 - Other and unspecified hyperlipidemia (17.0%, -3.7%), + V58.6 Long-term (current) drug use (16.2%, +26.4%), + 995.9 Systemic inflammatory response syndrome (SIRS) (14.9%, +81.6%), + 244.9 - Unspecified acquired hypothyroidism (13.7%, +85.6%), $\sim$ 530.8 Other specified disorders of esophagus (13.4%, +2.8%)	+ 518.8 Other disease of lung (29.2%, +118.1%), − 414.0 Coronary atherosclerosis (20.3%, -38.0%), − 600.0 Hypertrophy (benign) of prostate (1.0%, -82.9%), + 599.0 - Urinary tract infection, site not specified (18.2%, +84.1%), + 733.0 Osteoporosis (6.9%, +219.8%), + 785.5 Shock without mention of trauma (12.7%, +112.0%), + 995.9 Systemic inflammatory response syndrome (SIRS) (14.9%, +81.6%), + 038.9 - Unspecified septicemia (10.9%, +106.3%), + 244.9 - Unspecified acquired hypothyroidism (13.7%, +85.6%), + 294 Persistent mental disorders due to conditions classified elsewhere (6.6%, +174.6%), + 518.5 Pulmonary insufficiency following trauma and surgery (6.2%, +131.0%), + 276.2 - Acidosis (11.9%, +67.5%), + 507.0 - Pneumonitis due to inhalation of food or vomitus (9.9%, +72.4%)	+ 38.93 - Venous catheterization, not elsewhere classified (31.8%, +52.1%), + 96.04 - Insertion of endotracheal tube (26.7%, +144.9%), + 96.6 - Enteral infusion of concentrated nutritional substances (23.0%, +132.5%), + 96.71 - Continuous invasive mechanical ventilation for less than 96 consecutive hours (21.7%, +84.7%), + 96.72 - Continuous invasive mechanical ventilation for 96 consecutive hours or more (19.7%, +240.9%), − 99.04 - Transfusion of packed cells (13.0%, -9.0%), + 38.91 - Arterial catheterization (12.7%, +104.2%), + 99.15 - Parenteral infusion of concentrated nutritional substances (7.9%, +69.6%), − 39.61 - Extracorporeal circulation auxiliary to open heart surgery (7.6%, -56.0%), + 39.95 - Hemodialysis (7.3%, +26.8%), − 88.56 - Coronary arteriography using two catheters (7.1%, -42.0%), + 33.24 - Closed [endoscopic] biopsy of bronchus (7.0%, +68.0%), + 31.1 - Temporary tracheostomy (6.1%, +236.4%)	+ 96.04 - Insertion of endotracheal tube (26.7%, +144.9%), + 96.6 - Enteral infusion of concentrated nutritional substances (23.0%, +132.5%), + 96.72 - Continuous invasive mechanical ventilation for 96 consecutive hours or more (19.7%, +240.9%), − 36.15 - Single internal mammary-coronary artery bypass (3.9%, -67.4%), − 39.61 - Extracorporeal circulation auxiliary to open heart surgery (7.6%, -56.0%), + 96.71 - Continuous invasive mechanical ventilation for less than 96 consecutive hours (21.7%, +84.7%), + 38.93 - Venous catheterization, not elsewhere classified (31.8%, +52.1%), + 31.1 - Temporary tracheostomy (6.1%, +236.4%), + 38.91 - Arterial catheterization (12.7%, +104.2%), + 43.11 - Percutaneous [endoscopic] gastrostomy [PEG] (5.1%, +198.1%), − 36.12 - (Aorto)coronary bypass of two coronary arteries (1.8%, -64.2%), − 88.56 - Coronary arteriography using two catheters (7.1%, -42.0%), − 37.22 - Left heart cardiac catheterization (4.0%, -49.4%)
Cluster 2	N = 30452 22.3% female Age: 64 years (52, 76) ICU stay - all: 2 days (1, 4) - survivors: 2 days (1, 4) - deceased: 3 days (1, 6) 28-day mortality: 7.3%	$\sim$ 401.9 - Unspecified essential hypertension (41.9%, +4.0%), + 414.0 Coronary atherosclerosis (32.7%, +61.4%), $\sim$ 427.3 Atrial fibrillation and flutter (26.2%, -3.8%), − 428.0 - Congestive heart failure, unspecified (24.7%, -13.1%), $\sim$ 250.0 Diabetes mellitus without mention of complication or manifestation classifiable to 250.1-250.9 (19.6%, -1.3%), $\sim$ 272.4 - Other and unspecified hyperlipidemia (17.7%, +3.8%), − 584.9 - Acute kidney failure, unspecified (16.4%, -22.1%), + 272.0 - Pure hypercholesterolemia (13.8%, +56.6%), + V45.8 Other postprocedural status (13.5%, +30.4%), − 518.8 Other disease of lung (13.4%, -54.1%), $\sim$ 530.8 Other specified disorders of esophagus (13.0%, -2.7%), − V58.6 Long-term (current) drug use (12.8%, -20.9%), $\sim$ 403.9 Unspecified hypertensive kidney disease (12.0%, -2.9%)	− 518.8 Other disease of lung (13.4%, -54.1%), + 414.0 Coronary atherosclerosis (32.7%, +61.4%), + 600.0 Hypertrophy (benign) of prostate (5.6%, +485.5%), − 599.0 - Urinary tract infection, site not specified (9.9%, -45.7%), − 733.0 Osteoporosis (2.2%, -68.7%), − 785.5 Shock without mention of trauma (6.0%, -52.8%), − 995.9 Systemic inflammatory response syndrome (SIRS) (8.2%, -44.9%), − 038.9 - Unspecified septicemia (5.3%, -51.5%), − 244.9 - Unspecified acquired hypothyroidism (7.4%, -46.1%), − 294 Persistent mental disorders due to conditions classified elsewhere (2.4%, -63.6%), − 518.5 Pulmonary insufficiency following trauma and surgery (2.7%, -56.7%), − 276.2 - Acidosis (7.1%, -40.3%), − 507.0 - Pneumonitis due to inhalation of food or vomitus (5.7%, -42.0%)	− 38.93 - Venous catheterization, not elsewhere classified (20.9%, -34.3%), + 39.61 - Extracorporeal circulation auxiliary to open heart surgery (17.3%, +127.3%), + 99.04 - Transfusion of packed cells (14.3%, +9.9%), + 88.56 - Coronary arteriography using two catheters (12.2%, +72.4%), + 36.15 - Single internal mammary-coronary artery bypass (11.9%, +206.4%), − 96.71 - Continuous invasive mechanical ventilation for less than 96 consecutive hours (11.7%, -45.9%), − 96.04 - Insertion of endotracheal tube (10.9%, -59.2%), − 96.6 - Enteral infusion of concentrated nutritional substances (9.9%, -57.0%), + 37.22 - Left heart cardiac catheterization (7.9%, +97.6%), + 88.72 - Diagnostic ultrasound of heart (7.3%, +31.2%), − 38.91 - Arterial catheterization (6.2%, -51.0%), + 37.23 - Combined right and left heart cardiac catheterization (6.1%, +55.0%), − 96.72 - Continuous invasive mechanical ventilation for 96 consecutive hours or more (5.8%, -70.7%)	− 96.04 - Insertion of endotracheal tube (10.9%, -59.2%), − 96.6 - Enteral infusion of concentrated nutritional substances (9.9%, -57.0%), − 96.72 - Continuous invasive mechanical ventilation for 96 consecutive hours or more (5.8%, -70.7%), + 36.15 - Single internal mammary-coronary artery bypass (11.9%, +206.4%), + 39.61 - Extracorporeal circulation auxiliary to open heart surgery (17.3%, +127.3%), − 96.71 - Continuous invasive mechanical ventilation for less than 96 consecutive hours (11.7%, -45.9%), − 38.93 - Venous catheterization, not elsewhere classified (20.9%, -34.3%), − 31.1 - Temporary tracheostomy (1.8%, -70.3%), − 38.91 - Arterial catheterization (6.2%, -51.0%), − 43.11 - Percutaneous [endoscopic] gastrostomy [PEG] (1.7%, -66.4%), + 36.12 - (Aorto)coronary bypass of two coronary arteries (5.1%, +179.5%), + 88.56 - Coronary arteriography using two catheters (12.2%, +72.4%), + 37.22 - Left heart cardiac catheterization (7.9%, +97.6%)

We show basic descriptive statistics of the two clusters as well as the population. Frequent ICD-9 diagnoses or procedures are shown if they are enriched within a cluster (“+” for positively enriched, “−” for negatively enriched) and are sorted by the degree of enrichment within the cluster from high to low. “Prevalent” codes are sorted by the prevalence. Note that often, negatively enriched codes can be just as important in understanding a cluster as positively enriched codes. After each code, we show its support within the cluster (i.e. the fraction of admissions within the cluster that exhibit all diagnoses in the itemset) and the ratio between support in the cluster and support in the complement. Distributions of frequent ICD-9 codes vary between clusters. While patients in Cluster 1 suffer from acute conditions (lung diseases, heart failure, acute kidney failure, septicemia) more often, patients in Cluster 2 are more likely to exhibit chronic conditions. Additionally, differences between the clusters in terms of age and sex distribution, length of ICU stay, and mortality can be observed. Apart from age and sex, none of this data is supplied as input to our model. Thus, it follows these differences stem from information captured in the learned feature space.

**Figure 3:**
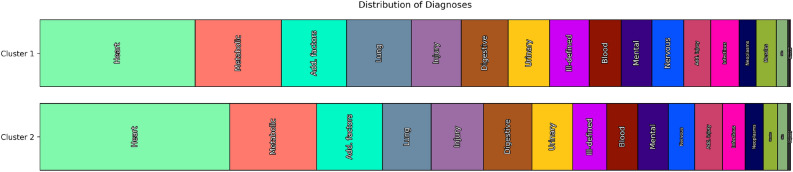
Distribution of ICD categories within the coarse clustering ($k=2$). For each clustering, we examine every diagnosis code assigned to any patient and count it towards the ICD group to which it belongs. Every diagnosis is counted, even if a single admission exhibits multiple diagnoses from the same ICD category. Typically, patients suffer from conditions in multiple ICD categories, so most patients contribute to multiple categories. Note that total diagnosis counts are different between clusters, but the bars in the figure are normalized to the same length to enable an easier comparison.

We do not supply information about the mortality or diagnoses to our model during training or inference. It is only used for evaluating clustering results. Thus, patterns observed in the clusters originate from the patients’ age, sex, and dynamic data.

**Figure 4:**
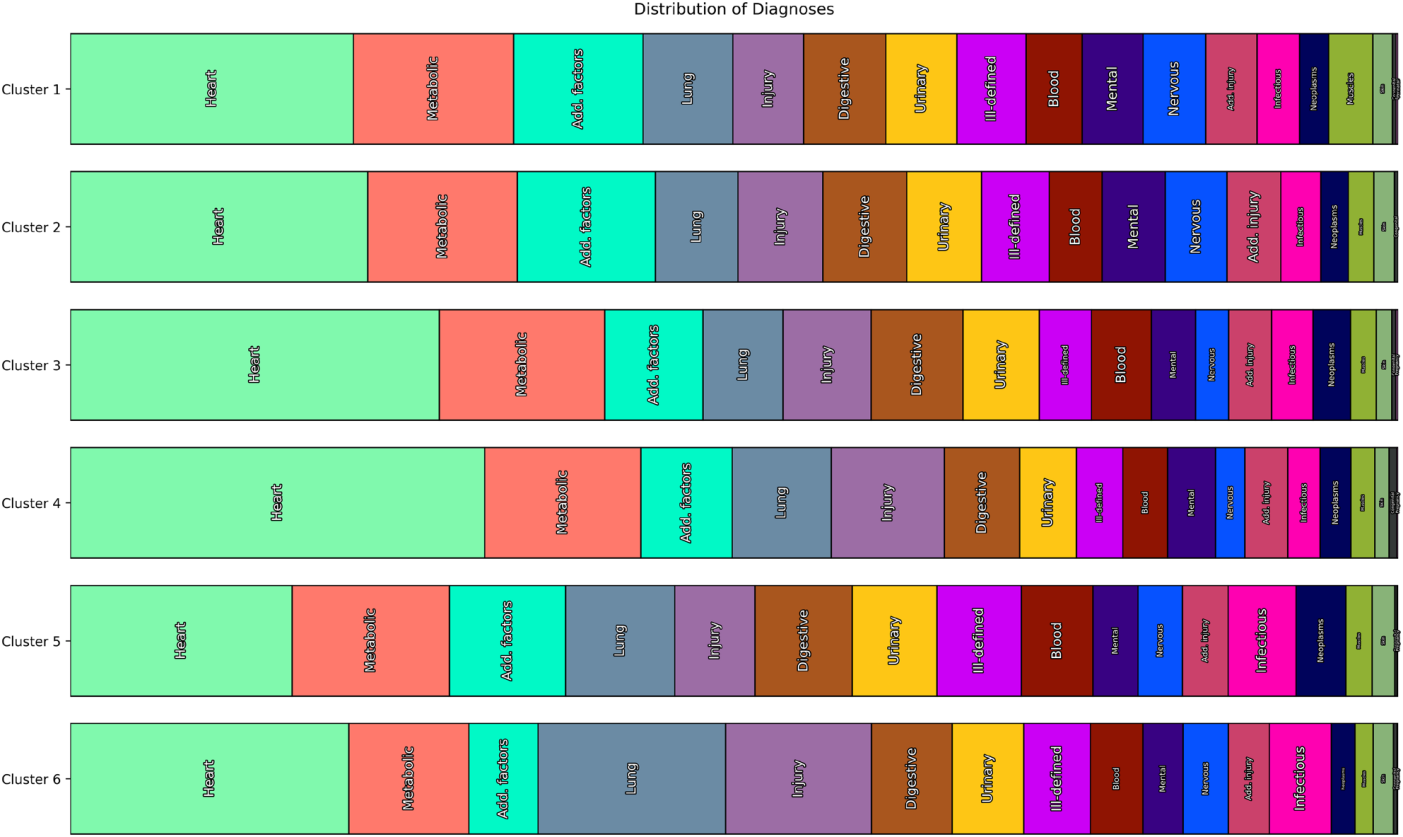
Distribution of ICD categories within the primary clustering ($k=6$). For each clustering, we examine every diagnosis code assigned to any patient and count it towards the ICD group to which it belongs. Every diagnosis is counted, even if a single admission exhibits multiple diagnoses from the same ICD category. Clusters 1 and 2 appear very similar concerning ICD categories; they differ more strongly on lower levels of the ICD tree. Cluster 4 has the highest fraction of heart-related diagnoses, while Cluster 6 contains the most injuries. Note that total diagnosis counts are different between clusters, but the bars in the figure are normalized to the same length to enable an easier comparison.

**Figure 5:**
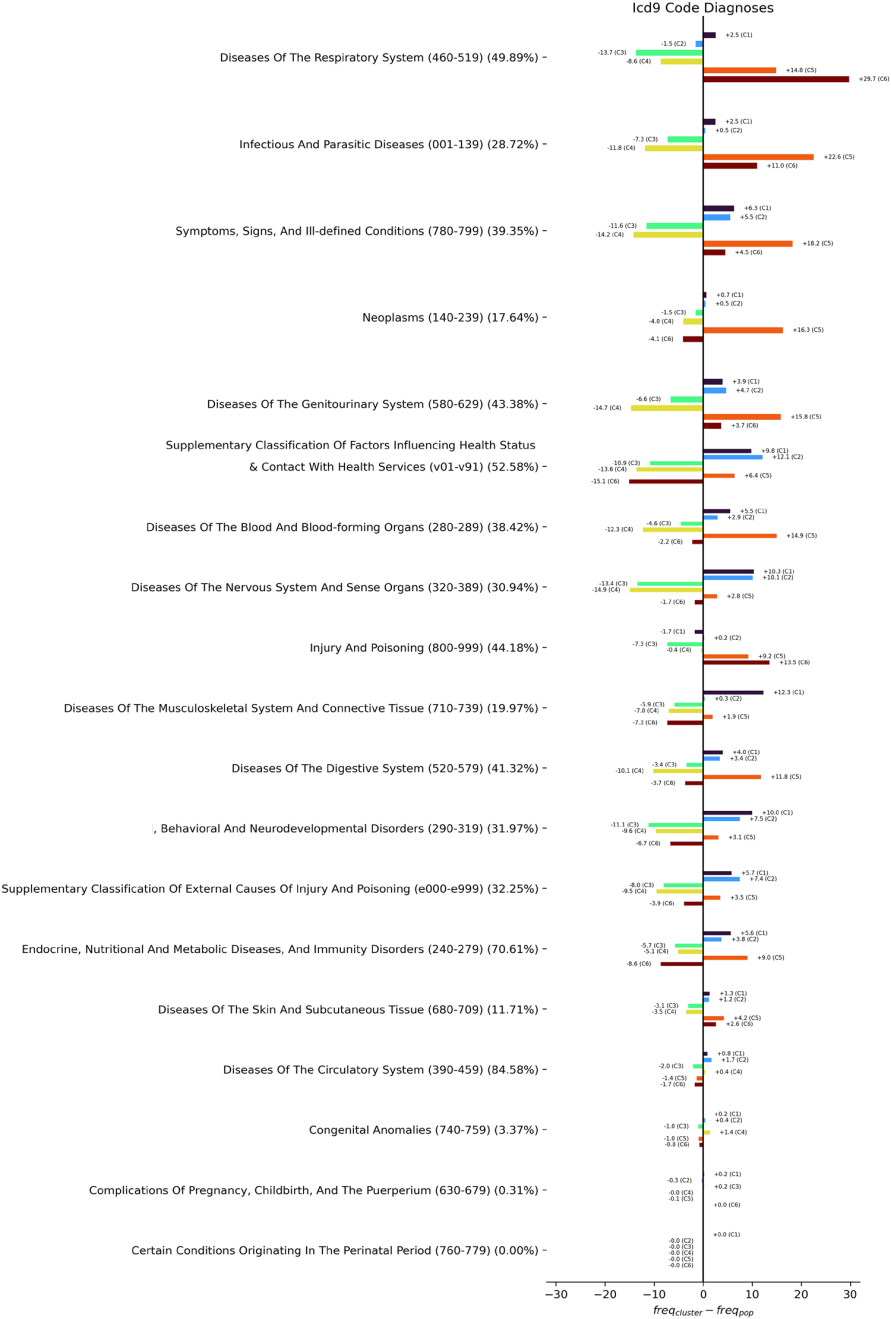
Differences of distribution of ICD categories within the primary clustering ($k=6$). For every ICD category, we measure the frequency within the entire population (denoted in parentheses after the category name) and then plot the difference in frequency in different clusters compared to the population frequency. Categories are sorted by the magnitude of the largest difference found within any clusters for the category. Note that since patients may have many diagnoses, clusters can have a higher frequency than the population for many categories (e.g. Cluster 5).

In the following, we first discuss the results of the coarse clustering (with $k=2$, Table 1) and then the primary clustering ($k=6$, Table 2). Note that in the table columns for enriched codes (diagnoses and procedures), only positively or negatively enriched codes are shown, not those that appear within the clusters with about the same frequency as within the cluster’s complement. We show bar plots comparing the ICD diagnosis category distribution between the clusters in Fig. 3 (for $k=2$) and Fig. 4 ($k=6$). For the primary clustering, we offer a tornado plot showing the differences in ICD disease category distribution between clusters (Fig. 5). These figures enable a quick visual comparison between diagnosis distribution in clusters. They were created by iterating over all patients within a cluster, examining each of their ICD diagnoses, and then counting the respective ICD top-level categories (e.g., *460–519: Diseases of the Respiratory System*, which becomes “Lung” in the figures). Every diagnosis is counted, even multiple ones for the same patient.

Since the coarse clustering splits the population into only two groups, the two resulting groups of patients are relatively large and still quite heterogeneous. However, it is noteworthy that Cluster 1 and 2 differ mainly in terms of the distribution of sex ($78\%$ female vs. $22\%$ female) and mortality ($23.5\%$ vs. $7.3\%$). In contrast, the length of stay on ICU and age distribution are relatively similar between the two clusters. As expected from the considerably different mortality rates alone, Cluster 1, i.e., the cluster with the higher mortality rate, was more likely to contain acute diagnoses, which are generally associated with greater severity of illness. These enriched diagnoses encompass two greater groups, namely diagnoses associated with sepsis (*038.9*; *995.9*) and its complications (*785.5*, *584.5* and *584.9*, *276.2*), as well as diagnoses affecting the pulmonary system with at least partial requirement for mechanical ventilation (*518.5*, *518.8*, *507.0*, *486*). The disease categories of infectious and parasitic diseases and diseases of the respiratory system are over-represented in Cluster 1 ($40.7\%$ and $45.3\%$ more likely in Cluster 1 than in Cluster 2), and these categories also carry with them high mortality: $28.2\%$ and $27.1\%$ in Cluster 1, $12\%$ and $11.2\%$ in Cluster 2. These findings are congruent with the list of procedures conducted in the ICU which shows a clear enrichment of procedures associated with partly long-term mechanical ventilation (*96.72* and *96.71*, *31.1*, *96.04*) and special nutritional procedures indicating that an independent oral nutrition intake was not possible (*43.11*, *96.6*, *99.15*). Cluster 2, in contrast, shows enrichment of cardiac diseases (*414.0*, *997.1*, *412*, *424.1*, *424.0*, *410.7*) and the respective procedures (*36.15* and *36.12*, *39.61*, *88.56*, *37.22*, *88.53*, and *37.23*). Other enrichment phenomena derive from the unequal sex distribution between the two clusters. This affects diagnoses with a sex-specific distribution like Hypertrophy of the prostate (*600.0*) or Osteoporosis (*733.0*). Looking at the absolute prevalence of diagnoses in the two clusters, the picture is much less clear. Thus, among the 12 most frequently occurring diagnoses in both clusters, nine diagnoses occur among the Top 12 in both clusters, just with a different order of frequency. These frequent diagnoses include typical endemic diseases like Essential hypertension, Coronary atherosclerosis and Diabetes mellitus. Neoplasms, which form the most lethal category of diagnoses, with $21\%$ of admissions ending with the patient’s death within 28 days of admission, are almost evenly distributed over the two clusters (taking their size difference into account). However, patients with neoplasm from Cluster 1 have a mortality of $30.5\%$, which is more than double that of those in Cluster 2 with the same diagnosis ($14.7\%$) It can therefore be concluded that it is not so much the most frequent diagnoses in absolute terms that lead to the marked difference in the outcome but other factors whose distribution differs across the clusters. In summary, patients in Cluster 1 are more likely to suffer from largely fatal conditions than those in Cluster 2, and this effect compounds over multiple disease categories.

**Table 2:** Primary clustering results.

Primary cluster name	Descriptive statistics	Prevalent diagnoses	Enriched diagnoses	Prevalent procedures	Enriched procedures
Cluster 1	N = 11174 99.5% female Age: 70 years (56, 82) ICU stay - all: 2 days (1, 4) - survivors: 2 days (1, 4) - deceased: 3 days (1, 6) 28-day mortality: 13.6%	+ 401.9 - Unspecified essential hypertension (43.8%, +7.8%), $\sim$ 428.0 - Congestive heart failure, unspecified (27.7%, +7.6%), $\sim$ 427.3 Atrial fibrillation and flutter (27.4%, +4.0%), + 272.4 - Other and unspecified hyperlipidemia (22.9%, +44.2%), − 414.0 Coronary atherosclerosis (22.4%, -24.4%), + 599.0 - Urinary tract infection, site not specified (20.3%, +84.4%), $\sim$ 250.0 Diabetes mellitus without mention of complication or manifestation classifiable to 250.1-250.9 (20.2%, +3.7%), $\sim$ 518.8 Other disease of lung (19.7%, +1.3%), + V58.6 Long-term (current) drug use (19.5%, +55.2%), $\sim$ 584.9 - Acute kidney failure, unspecified (19.3%, +7.5%), + 244.9 - Unspecified acquired hypothyroidism (16.9%, +116.5%), + 530.8 Other specified disorders of esophagus (16.8%, +38.9%), + 285.9 - Anemia, unspecified (13.6%, +37.7%)	+ 733.0 Osteoporosis (9.5%, +302.5%), − 600.0 Hypertrophy (benign) of prostate (0.1%, -98.4%), + 244.9 - Unspecified acquired hypothyroidism (16.9%, +116.5%), + V10.3 - Personal history of malignant neoplasm of breast (6.3%, +328.8%), + 599.0 - Urinary tract infection, site not specified (20.3%, +84.4%), + 428.3 Diastolic heart failure (12.6%, +114.4%), + 294 Persistent mental disorders due to conditions classified elsewhere (7.9%, +172.7%), + 311 - Depressive disorder, not elsewhere classified (11.4%, +110.1%), + 300.0 Anxiety states (6.4%, +152.2%), + V49.8 Other specified conditions influencing health status (6.4%, +147.9%), + V58.6 Long-term (current) drug use (19.5%, +55.2%), + 272.4 - Other and unspecified hyperlipidemia (22.9%, +44.2%), + V12.5 Personal history of diseases of circulatory system (9.4%, +81.7%)	− 38.93 - Venous catheterization, not elsewhere classified (23.3%, -9.2%), $\sim$ 96.71 - Continuous invasive mechanical ventilation for less than 96 consecutive hours (14.9%, -5.6%), − 96.04 - Insertion of endotracheal tube (13.4%, -25.7%), − 96.6 - Enteral infusion of concentrated nutritional substances (13.1%, -15.0%), − 39.61 - Extracorporeal circulation auxiliary to open heart surgery (8.6%, -42.8%), − 96.72 - Continuous invasive mechanical ventilation for 96 consecutive hours or more (8.5%, -29.1%), $\sim$ 38.91 - Arterial catheterization (8.1%, -8.7%), − 99.04 - Transfusion of packed cells (7.8%, -49.7%), − 88.56 - Coronary arteriography using two catheters (6.9%, -38.3%), $\sim$ 39.95 - Hemodialysis (5.9%, -8.7%), + 38.97 - Central venous catheter placement with guidance (5.8%, +172.3%), $\sim$ 33.24 - Closed [endoscopic] biopsy of bronchus (4.7%, -13.0%), $\sim$ 45.13 - Other endoscopy of small intestine (4.4%, -18.5%)	− 99.04 - Transfusion of packed cells (7.8%, -49.7%), − 36.15 - Single internal mammary-coronary artery bypass (4.1%, -59.8%), + 38.97 - Central venous catheter placement with guidance (5.8%, +172.3%), − 36.0 Removal of coronary artery obstruction and insertion of stent(s) (0.4%, -86.3%), − 39.61 - Extracorporeal circulation auxiliary to open heart surgery (8.6%, -42.8%), − 36.12 - (Aorto)coronary bypass of two coronary arteries (1.7%, -61.4%), − 99.20 - Injection or infusion of platelet inhibitor (1.4%, -63.5%), − 88.53 - Angiocardiography of left heart structures (2.0%, -57.5%), − 88.56 - Coronary arteriography using two catheters (6.9%, -38.3%), − 36.13 - (Aorto)coronary bypass of three coronary arteries (1.3%, -63.4%), − 99.07 - Transfusion of other serum (2.0%, -56.3%), − 99.05 - Transfusion of platelets (0.9%, -66.1%), − 37.23 - Combined right and left heart cardiac catheterization (3.2%, -46.5%)
Cluster 2	N = 12823 0.2% female Age: 65 years (53, 77) ICU stay - all: 2 days (1, 4) - survivors: 2 days (1, 4) - deceased: 3 days (1, 6) 28-day mortality: 11.7%	$\sim$ 401.9 - Unspecified essential hypertension (41.0%, -1.0%), + 414.0 Coronary atherosclerosis (31.6%, +18.3%), + 272.4 - Other and unspecified hyperlipidemia (27.9%, +102.1%), $\sim$ 427.3 Atrial fibrillation and flutter (27.8%, +6.3%), − 428.0 - Congestive heart failure, unspecified (24.4%, -9.2%), $\sim$ 250.0 Diabetes mellitus without mention of complication or manifestation classifiable to 250.1-250.9 (20.5%, +6.1%), + 584.9 - Acute kidney failure, unspecified (20.1%, +14.3%), + V58.6 Long-term (current) drug use (19.7%, +62.0%), + V45.8 Other postprocedural status (18.3%, +79.6%), − 518.8 Other disease of lung (18.1%, -9.3%), + 403.9 Unspecified hypertensive kidney disease (16.9%, +61.6%), + 530.8 Other specified disorders of esophagus (15.4%, +24.3%), + V15.8 Other specified personal history presenting hazards to health (11.7%, +93.3%)	+ 272.4 - Other and unspecified hyperlipidemia (27.9%, +102.1%), + 600.0 Hypertrophy (benign) of prostate (9.0%, +354.6%), − V10.3 - Personal history of malignant neoplasm of breast (0.1%, -97.7%), + 428.2 Systolic heart failure (10.9%, +133.4%), + V45.8 Other postprocedural status (18.3%, +79.6%), + 327.2 Organic sleep apnea (8.6%, +145.4%), + V58.6 Long-term (current) drug use (19.7%, +62.0%), + V15.8 Other specified personal history presenting hazards to health (11.7%, +93.3%), + 403.9 Unspecified hypertensive kidney disease (16.9%, +61.6%), + 585.9 - Chronic kidney disease, unspecified (10.4%, +82.3%), + 305.1 - Tobacco use disorder (9.9%, +79.2%), − 733.0 Osteoporosis (1.8%, -62.9%), + 274.9 - Gout, unspecified (6.7%, +104.2%)	− 38.93 - Venous catheterization, not elsewhere classified (21.2%, -20.0%), $\sim$ 96.71 - Continuous invasive mechanical ventilation for less than 96 consecutive hours (15.7%, +0.7%), $\sim$ 96.6 - Enteral infusion of concentrated nutritional substances (14.6%, -3.3%), $\sim$ 39.61 - Extracorporeal circulation auxiliary to open heart surgery (14.2%, +6.0%), − 96.04 - Insertion of endotracheal tube (13.4%, -26.3%), + 36.15 - Single internal mammary-coronary artery bypass (9.9%, +17.9%), − 96.72 - Continuous invasive mechanical ventilation for 96 consecutive hours or more (9.0%, -25.0%), − 88.56 - Coronary arteriography using two catheters (8.9%, -16.5%), − 38.91 - Arterial catheterization (7.5%, -17.6%), $\sim$ 39.95 - Hemodialysis (7.0%, +14.0%), − 99.04 - Transfusion of packed cells (6.3%, -61.7%), $\sim$ 37.22 - Left heart cardiac catheterization (6.1%, -6.8%), $\sim$ 33.24 - Closed [endoscopic] biopsy of bronchus (5.9%, +15.9%)	− 99.04 - Transfusion of packed cells (6.3%, -61.7%), − 36.0 Removal of coronary artery obstruction and insertion of stent(s) (0.3%, -90.6%), − 88.53 - Angiocardiography of left heart structures (1.9%, -61.0%), − 00.17 - Infusion of vasopressor agent (0.4%, -79.9%), − 99.05 - Transfusion of platelets (0.9%, -68.7%), − 99.07 - Transfusion of other serum (2.1%, -55.2%), − 37.23 - Combined right and left heart cardiac catheterization (3.1%, -47.9%), + 38.97 - Central venous catheter placement with guidance (4.7%, +99.9%), − 96.04 - Insertion of endotracheal tube (13.4%, -26.3%), − 38.93 - Venous catheterization, not elsewhere classified (21.2%, -20.0%), − 89.64 - Pulmonary artery wedge monitoring (0.9%, -63.2%), − 99.20 - Injection or infusion of platelet inhibitor (2.0%, -48.8%), − 36.06 - Insertion of non-drug-eluting coronary artery stent(s) (1.2%, -55.8%)
Cluster 3	N = 10472 46.5% female Age: 63 years (50, 76) ICU stay - all: 2 days (1, 3) - survivors: 2 days (1, 3) - deceased: 3 days (1, 5) 28-day mortality: 5.5%	$\sim$ 401.9 - Unspecified essential hypertension (39.8%, -4.6%), $\sim$ 414.0 Coronary atherosclerosis (28.4%, +2.1%), $\sim$ 428.0 - Congestive heart failure, unspecified (26.1%, -0.5%), − 427.3 Atrial fibrillation and flutter (21.9%, -21.3%), − 250.0 Diabetes mellitus without mention of complication or manifestation classifiable to 250.1-250.9 (17.8%, -12.0%), − 584.9 - Acute kidney failure, unspecified (15.7%, -17.3%), + 272.0 - Pure hypercholesterolemia (15.3%, +40.1%), − 530.8 Other specified disorders of esophagus (11.4%, -16.3%), + 276.5 Volume depletion (11.0%, +37.9%), − V45.8 Other postprocedural status (11.0%, -13.7%), − 599.0 - Urinary tract infection, site not specified (10.7%, -22.5%), − 403.9 Unspecified hypertensive kidney disease (10.1%, -20.4%), − 272.4 - Other and unspecified hyperlipidemia (9.5%, -51.2%)	− 518.8 Other disease of lung (7.1%, -68.9%), − V49.8 Other specified conditions influencing health status (0.2%, -95.3%), − 272.4 - Other and unspecified hyperlipidemia (9.5%, -51.2%), − 518.5 Pulmonary insufficiency following trauma and surgery (0.5%, -90.3%), − 276.0 - Hyperosmolality and/or hypernatremia (1.1%, -79.1%), − 507.0 - Pneumonitis due to inhalation of food or vomitus (3.0%, -65.0%), − 294 Persistent mental disorders due to conditions classified elsewhere (1.0%, -79.3%), − 785.5 Shock without mention of trauma (4.3%, -55.5%), − V58.6 Long-term (current) drug use (8.8%, -43.3%), − 995.9 Systemic inflammatory response syndrome (SIRS) (6.2%, -48.7%), − 276.2 - Acidosis (5.1%, -48.6%), − V12.5 Personal history of diseases of circulatory system (3.1%, -55.7%), − V66.7 - Encounter for palliative care (0.4%, -83.9%)	− 38.93 - Venous catheterization, not elsewhere classified (18.5%, -31.3%), + 99.04 - Transfusion of packed cells (18.2%, +43.2%), + 88.56 - Coronary arteriography using two catheters (13.1%, +38.9%), + 37.23 - Combined right and left heart cardiac catheterization (8.7%, +100.5%), + 99.20 - Injection or infusion of platelet inhibitor (8.3%, +303.8%), + 36.0 Removal of coronary artery obstruction and insertion of stent(s) (7.4%, +539.8%), $\sim$ 37.22 - Left heart cardiac catheterization (6.8%, +8.9%), + 45.13 - Other endoscopy of small intestine (6.7%, +39.5%), $\sim$ 39.95 - Hemodialysis (5.7%, -13.7%), + 36.06 - Insertion of non-drug-eluting coronary artery stent(s) (5.6%, +269.2%), − 39.61 - Extracorporeal circulation auxiliary to open heart surgery (5.5%, -65.2%), + 36.07 - Insertion of drug-eluting coronary artery stent(s) (5.1%, +230.9%), $\sim$ 88.53 - Angiocardiography of left heart structures (4.8%, +21.0%)	− 96.04 - Insertion of endotracheal tube (2.4%, -88.7%), − 96.71 - Continuous invasive mechanical ventilation for less than 96 consecutive hours (2.8%, -85.4%), − 96.6 - Enteral infusion of concentrated nutritional substances (3.1%, -82.9%), − 96.72 - Continuous invasive mechanical ventilation for 96 consecutive hours or more (0.5%, -96.4%), + 36.0 Removal of coronary artery obstruction and insertion of stent(s) (7.4%, +539.8%), − 39.61 - Extracorporeal circulation auxiliary to open heart surgery (5.5%, -65.2%), + 99.20 - Injection or infusion of platelet inhibitor (8.3%, +303.8%), − 38.97 - Central venous catheter placement with guidance (0.0%, -100.0%), − 31.1 - Temporary tracheostomy (0.2%, -94.5%), − 36.15 - Single internal mammary-coronary artery bypass (3.5%, -65.3%), − 33.24 - Closed [endoscopic] biopsy of bronchus (1.5%, -76.9%), + 36.06 - Insertion of non-drug-eluting coronary artery stent(s) (5.6%, +269.2%), − 43.11 - Percutaneous [endoscopic] gastrostomy [PEG] (0.4%, -89.9%)
Cluster 4	N = 7007 31.5% female Age: 64 years (53, 75) ICU stay - all: 2 days (1, 4) - survivors: 2 days (1, 4) - deceased: 3 days (2, 5) 28-day mortality: 2.9%	+ 401.9 - Unspecified essential hypertension (47.2%, +17.0%), + 414.0 Coronary atherosclerosis (42.2%, +64.7%), $\sim$ 427.3 Atrial fibrillation and flutter (27.8%, +5.4%), $\sim$ 428.0 - Congestive heart failure, unspecified (24.1%, -9.2%), + 272.0 - Pure hypercholesterolemia (21.3%, +106.5%), $\sim$ 250.0 Diabetes mellitus without mention of complication or manifestation classifiable to 250.1-250.9 (21.2%, +9.0%), − 518.8 Other disease of lung (14.4%, -29.3%), − 530.8 Other specified disorders of esophagus (11.5%, -14.8%), − 272.4 - Other and unspecified hyperlipidemia (10.8%, -41.6%), + 411.1 - Intermediate coronary syndrome (10.4%, +393.7%), + 997.1 - Cardiac complications, not elsewhere classified (10.2%, +171.6%), + 424.0 - Mitral valve disorders (9.6%, +83.4%), − 584.9 - Acute kidney failure, unspecified (9.3%, -52.8%)	+ 411.1 - Intermediate coronary syndrome (10.4%, +393.7%), + 414.0 Coronary atherosclerosis (42.2%, +64.7%), + 272.0 - Pure hypercholesterolemia (21.3%, +106.5%), − 584.9 - Acute kidney failure, unspecified (9.3%, -52.8%), − 995.9 Systemic inflammatory response syndrome (SIRS) (4.2%, -65.1%), + 997.1 - Cardiac complications, not elsewhere classified (10.2%, +171.6%), − V58.6 Long-term (current) drug use (6.8%, -55.8%), − V49.8 Other specified conditions influencing health status (0.2%, -95.0%), − 785.5 Shock without mention of trauma (3.6%, -61.6%), − 272.4 - Other and unspecified hyperlipidemia (10.8%, -41.6%), − 038.9 - Unspecified septicemia (3.1%, -62.0%), − 403.9 Unspecified hypertensive kidney disease (6.8%, -47.9%), − 294 Persistent mental disorders due to conditions classified elsewhere (1.1%, -76.1%)	+ 39.61 - Extracorporeal circulation auxiliary to open heart surgery (41.0%, +351.3%), + 36.15 - Single internal mammary-coronary artery bypass (28.4%, +410.7%), + 96.71 - Continuous invasive mechanical ventilation for less than 96 consecutive hours (22.9%, +59.5%), + 99.04 - Transfusion of packed cells (22.2%, +78.4%), − 38.93 - Venous catheterization, not elsewhere classified (20.5%, -20.7%), + 96.04 - Insertion of endotracheal tube (19.8%, +19.9%), + 88.56 - Coronary arteriography using two catheters (17.2%, +88.8%), + 88.72 - Diagnostic ultrasound of heart (14.2%, +165.3%), + 37.22 - Left heart cardiac catheterization (13.0%, +145.2%), + 36.12 - (Aorto)coronary bypass of two coronary arteries (12.9%, +459.5%), + 88.53 - Angiocardiography of left heart structures (12.2%, +332.7%), + 36.13 - (Aorto)coronary bypass of three coronary arteries (9.4%, +383.6%), − 96.6 - Enteral infusion of concentrated nutritional substances (9.1%, -42.9%)	+ 39.61 - Extracorporeal circulation auxiliary to open heart surgery (41.0%, +351.3%), + 36.15 - Single internal mammary-coronary artery bypass (28.4%, +410.7%), + 36.12 - (Aorto)coronary bypass of two coronary arteries (12.9%, +459.5%), + 88.53 - Angiocardiography of left heart structures (12.2%, +332.7%), + 36.13 - (Aorto)coronary bypass of three coronary arteries (9.4%, +383.6%), − 96.72 - Continuous invasive mechanical ventilation for 96 consecutive hours or more (3.4%, -72.5%), + 88.72 - Diagnostic ultrasound of heart (14.2%, +165.3%), + 36.11 - (Aorto)coronary bypass of one coronary artery (6.0%, +384.7%), + 37.22 - Left heart cardiac catheterization (13.0%, +145.2%), − 38.97 - Central venous catheter placement with guidance (0.0%, -100.0%), + 99.04 - Transfusion of packed cells (22.2%, +78.4%), + 35.21 - Open and other replacement of aortic valve with tissue graft (7.0%, +222.8%), + 88.56 - Coronary arteriography using two catheters (17.2%, +88.8%)
Cluster 5	N = 3119 42.6% female Age: 68 years (56, 81) ICU stay - all: 2 days (1, 5) - survivors: 2 days (1, 5) - deceased: 2 days (1, 5) 28-day mortality: 34.1%	− 401.9 - Unspecified essential hypertension (36.6%, -12.0%), + 518.8 Other disease of lung (32.9%, +76.9%), + 584.9 - Acute kidney failure, unspecified (31.3%, +79.9%), + 995.9 Systemic inflammatory response syndrome (SIRS) (29.9%, +213.7%), $\sim$ 427.3 Atrial fibrillation and flutter (27.5%, +3.7%), $\sim$ 428.0 - Congestive heart failure, unspecified (26.8%, +2.6%), + 785.5 Shock without mention of trauma (22.3%, +191.0%), + 038.9 - Unspecified septicemia (20.8%, +218.4%), + 276.2 - Acidosis (20.3%, +146.6%), $\sim$ 250.0 Diabetes mellitus without mention of complication or manifestation classifiable to 250.1-250.9 (19.5%, -0.9%), + 486 Pneumonia, organism unspecified (16.9%, +86.0%), − 414.0 Coronary atherosclerosis (16.8%, -41.4%), $\sim$ 272.4 - Other and unspecified hyperlipidemia (16.4%, -6.1%)	+ 995.9 Systemic inflammatory response syndrome (SIRS) (29.9%, +213.7%), + 038.9 - Unspecified septicemia (20.8%, +218.4%), + 785.5 Shock without mention of trauma (22.3%, +191.0%), + 276.2 - Acidosis (20.3%, +146.6%), + 518.8 Other disease of lung (32.9%, +76.9%), + 584.9 - Acute kidney failure, unspecified (31.3%, +79.9%), + E933.1 - Antineoplastic and immunosuppressive drugs causing adverse effects in therapeutic use (5.3%, +521.9%), − 414.0 Coronary atherosclerosis (16.8%, -41.4%), + V49.8 Other specified conditions influencing health status (8.6%, +177.5%), + 486 Pneumonia, organism unspecified (16.9%, +86.0%), + 038.4 Septicemia due to other gram-negative organisms (6.0%, +225.7%), + 287.5 - Thrombocytopenia, unspecified (11.9%, +108.2%), + 780.6 Fever and other physiologic disturbances of temperature regulation (5.9%, +196.7%)	+ 38.93 - Venous catheterization, not elsewhere classified (37.2%, +52.8%), + 96.04 - Insertion of endotracheal tube (23.1%, +39.3%), + 96.71 - Continuous invasive mechanical ventilation for less than 96 consecutive hours (22.1%, +45.7%), $\sim$ 96.6 - Enteral infusion of concentrated nutritional substances (17.0%, +14.7%), + 96.72 - Continuous invasive mechanical ventilation for 96 consecutive hours or more (14.2%, +29.9%), + 38.91 - Arterial catheterization (14.0%, +67.2%), $\sim$ 99.04 - Transfusion of packed cells (11.3%, -19.2%), + 99.15 - Parenteral infusion of concentrated nutritional substances (9.7%, +72.0%), + 33.24 - Closed [endoscopic] biopsy of bronchus (8.8%, +72.9%), $\sim$ 39.95 - Hemodialysis (8.2%, +30.8%), + 38.97 - Central venous catheter placement with guidance (7.0%, +157.5%), + 54.91 - Percutaneous abdominal drainage (5.4%, +96.9%), + 99.25 - Injection or infusion of cancer chemotherapeutic substance (5.1%, +577.0%)	− 39.61 - Extracorporeal circulation auxiliary to open heart surgery (2.5%, -82.3%), − 36.15 - Single internal mammary-coronary artery bypass (1.2%, -86.9%), + 99.25 - Injection or infusion of cancer chemotherapeutic substance (5.1%, +577.0%), + 38.93 - Venous catheterization, not elsewhere classified (37.2%, +52.8%), − 88.56 - Coronary arteriography using two catheters (4.3%, -59.6%), + 38.97 - Central venous catheter placement with guidance (7.0%, +157.5%), − 36.12 - (Aorto)coronary bypass of two coronary arteries (0.6%, -84.8%), − 36.13 - (Aorto)coronary bypass of three coronary arteries (0.4%, -86.8%), − 37.22 - Left heart cardiac catheterization (2.4%, -63.9%), + 38.91 - Arterial catheterization (14.0%, +67.2%), + 96.71 - Continuous invasive mechanical ventilation for less than 96 consecutive hours (22.1%, +45.7%), − 88.53 - Angiocardiography of left heart structures (1.2%, -71.9%), − 99.20 - Injection or infusion of platelet inhibitor (0.8%, -76.5%)
Cluster 6	N = 5004 43.9% female Age: 67 years (53, 78) ICU stay - all: 9 days (4, 17) - survivors: 12 days (6, 22) - deceased: 5 days (2, 10) 28-day mortality: 37.0%	+ 518.8 Other disease of lung (47.3%, +189.0%), − 401.9 - Unspecified essential hypertension (34.5%, -18.1%), + 428.0 - Congestive heart failure, unspecified (30.2%, +17.3%), $\sim$ 427.3 Atrial fibrillation and flutter (29.2%, +10.8%), + 584.9 - Acute kidney failure, unspecified (21.0%, +16.8%), + 507.0 - Pneumonitis due to inhalation of food or vomitus (18.3%, +200.6%), $\sim$ 250.0 Diabetes mellitus without mention of complication or manifestation classifiable to 250.1-250.9 (18.2%, -8.4%), + 785.5 Shock without mention of trauma (18.1%, +141.4%), + 995.9 Systemic inflammatory response syndrome (SIRS) (17.8%, +78.0%), − 414.0 Coronary atherosclerosis (17.1%, -41.2%), + 599.0 - Urinary tract infection, site not specified (15.8%, +23.3%), + 518.5 Pulmonary insufficiency following trauma and surgery (15.7%, +470.7%), + 486 Pneumonia, organism unspecified (15.6%, +75.9%)	+ 518.8 Other disease of lung (47.3%, +189.0%), + 518.5 Pulmonary insufficiency following trauma and surgery (15.7%, +470.7%), − 272.4 - Other and unspecified hyperlipidemia (5.0%, -73.6%), + 482.4 Pneumonia due to Staphylococcus (8.5%, +600.8%), + 507.0 - Pneumonitis due to inhalation of food or vomitus (18.3%, +200.6%), + 785.5 Shock without mention of trauma (18.1%, +141.4%), + 038.9 - Unspecified septicemia (15.1%, +129.1%), + 427.5 - Cardiac arrest (7.7%, +252.8%), − 414.0 Coronary atherosclerosis (17.1%, -41.2%), + 584.5 - Acute kidney failure with lesion of tubular necrosis (10.3%, +162.0%), − 530.8 Other specified disorders of esophagus (5.8%, -58.7%), − V49.8 Other specified conditions influencing health status (0.2%, -95.3%), + 995.9 Systemic inflammatory response syndrome (SIRS) (17.8%, +78.0%)	+ 96.04 - Insertion of endotracheal tube (56.9%, +355.1%), + 96.72 - Continuous invasive mechanical ventilation for 96 consecutive hours or more (54.1%, +751.5%), + 38.93 - Venous catheterization, not elsewhere classified (52.2%, +136.1%), + 96.6 - Enteral infusion of concentrated nutritional substances (51.5%, +376.6%), + 96.71 - Continuous invasive mechanical ventilation for less than 96 consecutive hours (29.3%, +109.1%), + 99.04 - Transfusion of packed cells (27.3%, +121.7%), + 38.91 - Arterial catheterization (23.1%, +224.9%), + 31.1 - Temporary tracheostomy (18.7%, +947.0%), + 99.15 - Parenteral infusion of concentrated nutritional substances (18.2%, +299.9%), + 33.24 - Closed [endoscopic] biopsy of bronchus (14.4%, +236.0%), + 43.11 - Percutaneous [endoscopic] gastrostomy [PEG] (13.2%, +614.7%), + 99.07 - Transfusion of other serum (10.4%, +219.5%), + 88.72 - Diagnostic ultrasound of heart (10.4%, +67.4%)	+ 38.93 - Venous catheterization, not elsewhere classified (52.2%, +136.1%), + 31.1 - Temporary tracheostomy (18.7%, +947.0%), + 96.72 - Continuous invasive mechanical ventilation for 96 consecutive hours or more (54.1%, +751.5%), + 96.04 - Insertion of endotracheal tube (56.9%, +355.1%), + 96.6 - Enteral infusion of concentrated nutritional substances (51.5%, +376.6%), + 43.11 - Percutaneous [endoscopic] gastrostomy [PEG] (13.2%, +614.7%), + 38.91 - Arterial catheterization (23.1%, +224.9%), + 99.15 - Parenteral infusion of concentrated nutritional substances (18.2%, +299.9%), + 99.04 - Transfusion of packed cells (27.3%, +121.7%), + 96.71 - Continuous invasive mechanical ventilation for less than 96 consecutive hours (29.3%, +109.1%), + 33.24 - Closed [endoscopic] biopsy of bronchus (14.4%, +236.0%), + 00.17 - Infusion of vasopressor agent (7.1%, +574.6%), + 89.64 - Pulmonary artery wedge monitoring (7.5%, +389.8%)

Large differences between the clusters in terms of age distribution, length of ICU stay, and mortality can be observed. Distributions of ICD-9 diagnoses vary strongly between clusters. (For information about the population, please refer to Table 1).

The different distribution of certain diseases, degrees of severities and mortality, which was already indicated in the subdivision into two clusters, is now even more evident with a more detailed subdivision into six primary clusters, as the enriched frequent codes show in Table 2. Particularly, we compare the frequency of specific diagnoses within a cluster to its complement, i.e., to all admissions not in the cluster.

In the following, we give a short description of the resulting clusters:

Cluster 1 consists of the oldest patients (70 years), who are almost exclusively female. With about 11000 admissions, it is the second-largest cluster. The striking imbalance concerning patient sex is responsible for the enrichment of several diagnoses, which are quite typical for female patients of higher age. This includes diagnoses like osteoporosis (*733.0*), acquired hypothyroidism (*244.9*), urinary tract infection (*599.0*), diastolic heart failure (*428.3*), and mental conditions (*294*, *311*, and *300.0*), but also sex-specific neoplasms (*V10.3*). In contrast, typical diseases of men are underrepresented (*600.0*). Apart from minor shifts due to the sex imbalance, the absolute prevalences of diagnoses within the cluster differ only insignificantly from the prevalences of the total population, resulting in a clear focus on cardiovascular diseases and its complications like essential hypertension (*401.9*), congestive or diastolic heart failure (*428.0*, *428.3*), atrial fibrillation/flutter (*427.3*), or hypertensive kidney disease (*403.9*). The documented procedures do not show a clear picture since almost no procedure is enriched in this cluster. Although the cluster has both the highest age and diagnoses indicative of a certain degree of disease severity among those affected, mortality within the cluster does not differ from that of the overall population.

Cluster 2 can be seen as the counterpart to Cluster 1 since it contains nearly exclusively male patients. It is the largest cluster, with about 13 thousand admissions. The patients in this cluster are slightly younger than those in Cluster 1, but the first two clusters are largely similar regarding the length of stay and mortality. Again, the enrichment of diagnoses is mainly caused by the male predominance in this cluster. Thus, typical male diagnoses occur more frequently, like hypertrophy of the prostate (*600.0*), sleep apnea syndrome (*327.2*), gout (*274.9*) and tobacco use disorder (*305.1*). The ratio of female patients for diastolic heart failure is higher, while in systolic heart failure, the opposite is true. This can be seen in Cluster 1 and 2. Remarkably, the patients in Cluster 2 were more frequently diagnosed with coronary atherosclerosis (*414.0*) compared to Cluster 1. Hyperlipidemia (*272.4*), a highly relevant risk factor for developing this disease, occurs in this cluster more than twice as often as in the other clusters. Consequently, this results in a slight enrichment of coronary bypass procedures (*36.13*, *36.15*) treating this condition. Except for the codes mentioned above and similarly to Cluster 1, the diagnoses and procedures of this cluster do not differ relevantly from the total population.

Cluster 3 contains the youngest patient population (63 years), which shows relatively low mortality ($5.5\%$). The distribution of men and women and the most frequent diagnoses correspond to that of the total population. However, based on the enrichment analysis, it is noticeable that Cluster 3 is characterized by numerous diagnoses occurring less frequently than in other clusters. These also encompass very severe diagnoses, like pulmonary insufficiency (*518.5*), shock (*785.5*) and systemic inflammatory response syndrome (*995.9*), which can contribute to a prolonged length of stay and increased mortality. Also, the most frequent diagnoses in this cluster, like hypertension, acute atrial fibrillation, diabetes mellitus, or acute kidney failure, occur less frequently than in other clusters. Concerning the performed procedures, it can be assumed that a relevant proportion of patients were treated with a cardiac catheterization partly with the insertion of one or more stents into the coronary arteries (*36.0*, *36.06*, *36.07*, *37.22*, *37.23*, *88.53*, *88.56*). Although the absolute counts for the single interventions are quite low, they are significantly enriched in this cluster. For instance, the procedure “removal of coronary artery obstruction and insertion of stent(s)” (*36.0*) was conducted in $7.4\%$ of the cluster patients but more than six times more frequent ($+639.8\%$) than in the other clusters.

The patients in Cluster 4 have the lowest mortality of all clusters ($2.9\%$). The median age is two years lower than within the population (64*y* vs. 66*y*), and the cluster has a slight male predominance. In the synopsis of procedures and diagnoses, it becomes clear that this cluster consists of patients who underwent a cardiac procedure, partly interventional, partly surgical. Thus, codes representing different kinds of cardiac catheterization are highly enriched (*88.53*, *37.22*, *88.56*, *37.23*). Surprisingly, procedures with stent implantation (*36.0*, *36.06*, *36.07*) are underrepresented in this cluster. Additionally, different procedures of cardiac surgery, like coronary artery bypass surgery (*36.12*, *36.13*, *36.11*, *36.15*) or heart valve replacement (*35.21*) are highly enriched, with an up to 5.6-fold higher probability in this cluster. Also, procedures that are typically associated with cardiac surgery are highly enriched, like extracorporeal circulation during surgery (*39.61*), cardiac ultrasound (*88.72*), transfusion of different blood products (*99.04*, *99.05*, *99.07*) or short-term mechanical ventilation (*96.71*). This interpretation is supported by the clear enrichment of diagnoses that affect the coronary arteries (*411.1*, *413.9*, *414.0*, *410.7*) or the heart valves (*424.0*, *424.1*). Procedures that occur in the context of cardiac surgery, but indicate the occurrence of surgery-related complications, like a temporal tracheostomy (*31.1*) or hemodialysis (*39.95*), occur significantly less in this cluster. Due to the very low mortality, it can be assumed that the patients in this cluster develop serious complications only to a minimal extent.

Although similar in biometric features compared to previous clusters, the patients in Cluster 5, the smallest cluster with 3119 admissions, exhibit a relevantly increased mortality ($34.1\%$). There are prevalent diagnoses associated with sepsis, a life-threatening condition that arises when the body’s response to infection damages its tissues and organs. In the enrichment analysis, there are almost exclusively diagnoses representing the sepsis itself (*038.9*, *038.4*) or its symptoms, like systemic inflammatory response syndrome or fever (*995.9*, *780.6*), their triggering infectious conditions, like pneumonia (*486*), and finally its complications, like shock, acidosis, acute kidney failure or thrombocytopenia (*785.5*, *276.2*, *584.9*, *287.5*). Also highly enriched is iatrogenic immunosuppression through immunosuppressive or antineoplastic drugs (*E933.1*), which increases the risk for sepsis. Moving beyond the enriched diagnoses listed in Table 2, there is a multitude of additional sepsis triggers, like intestinal infection (*008.4*), aspirational pneumonitis (*507.0*), urinary tract infection (*599.0*) or infection of an internal prosthetic device, implant, or graft (*996.6*). The cornerstone of sepsis therapy consists of sufficient intravenous fluid replacement and therapy with broad-spectrum antibiotics. If a focus of infection is evident, it needs to be controlled, usually by draining the infectious fluids. Such a measure of focus control is the percutaneous abdominal drainage (*54.91*), which is enriched in this cluster. Apart from that, only a few interventions occur more frequently in this cluster than in its complement. Among them, there are central venous catheterizations for fluid replacement (*38.97*, *38.93*), arterial catheterizations (*38.91*), and measures to treat organ dysfunctions, like mechanical ventilation (*86.04*, *96.71*, *96.72*), hemodialysis (*38.95*, *39.95*) and administration of vasopressive agents (*00.17*).

Finally, Cluster 6 represents the cluster with the highest mortality of $37.0\%$, which is almost three times as high as within the total population. Moreover, it is the cluster with by far the most extended treatment duration in the ICU, namely 9 days in the median. The patients exhibit a clear gap in the length of stay between survivors and deceased patients (12 days vs. 9 days), indicating a prolonged recovery time if a life-threatening condition was survived. In the list of the most prevalent diagnosis as well as in the list of enriched diagnoses, lung-related problems, such as pneumonia/pneumonitis (*507.0*, *482.4*, *486*) or pulmonary insufficiency (*518.5*), are dominant. The most prevalent and most enriched diagnosis “*518.8* - Other diseases of the lung” ($47.3\%$, $+189.0\%$) contains different forms of acute and chronic respiratory failures, but also the acute respiratory distress syndrome (ARDS), a syndrome with a mortality up to $40\%$, which does not yet have a specific code in ICD-9. This clear focus on pulmonary diseases also results in an extraordinarily high rate of patients requiring mechanical ventilation, which applies to $84.4\%$ of patients (*96.71* - $29.3\%$ and *96.72* - $54.1\%$). Not only pulmonary diseases but also other severe conditions requiring mechanical ventilation, such as sepsis (*038.9*, *995.9*), shock (*785.5*) and congestive heart failure (*428.0*), occur more frequently in this cluster. The corresponding procedures are suggestive for patients with a long-term critical status requiring interventions according to the circumstances. These measures were carried out almost exclusively on patients in this cluster, resulting in substantial enrichment metrics. For instance, a percutaneous gastrostomy (*43.11*) for a long-term nutrition of a patient is carried out in $13.2\%$ of the patients in Cluster 6, while this applies to $1.9\%$ of the remaining clusters only. Similar rates can be found for a temporal tracheostomy (*31.1*) ($18.7\%$ in Cluster 6 vs. $1.8\%$ in clusters 1 to 5), resulting in a nearly 10-fold higher proportion. Additionally, other strongly enriched interventions, like enteral or parenteral nutrition (*96.6*, *99.15*), transfusion of different blood products (*99.05*, *99.07*, *99.04*), application of vasopressors (*00.17*) give a strong indication of the critical status of the patients and requirement of long-term treatment in this cluster, since these measures would usually not be necessary for a less severely affected population.

In summary, we performed two clusterings: A coarse clustering with 2 clusters and a finer one with 6 clusters. The coarse clustering already shows clear differences in mortality and diagnosis distribution, especially regarding specific diagnoses as opposed to ICD categories. The primary clustering shows more homogeneous groups of patients with different disease patterns, such as severe lung problems, sepsis, or heart disease.

## Discussion

In this work, we developed an autoencoder model trained on dynamic clinical data with time as an additional input. Using positional encodings inspired by Vaswani et al.^27^ to express time allowed the model to reconstruct the time series without relying on a temporal grid of fixed size, resulting in an $18\%$ reduction in reconstruction error when compared with a simple time representation. Note that the positional encodings do not add any information not already available to the model since the time is also supplied as the number of minutes since the start of the admission. However, the positional encodings are a representation of time that is more intuitive for the GRU model to learn and reason with, and thus leads to better reconstruction than just expressing time in minutes. Our finding demonstrates that representations can be powerful tools to augment the capabilities of machine learning models.

By clustering on the learned feature space at multiple scales, we were able to identify clinically meaningful clusters: They exhibit patterns also observed in clinical practice, such as sepsis or severe lung problems. These patterns observed in ICD diagnoses were learned without giving the model access to the diagnoses.

However, the discovered clusters are not pure, i.e., there are no clusters that only contain a single disease class. Additionally, we only include the 75 dynamic data attributes measured for at least $50\%$ of the population, so many laboratory results used in clinical practice (especially those that make up the “long tail” of rare attributes) are not accessible to our model, even though these could be extremely informative. Another limitation is that the model sometimes fails to capture the temporal characteristics of time series (for examples, see our supplementary materials).

Due to the advantage of being able to deal with longitudinal data, we chose an autoencoder to examine the database for meaningful clusters. We focused on dynamic patient data supplemented with a minimum of static data like sex and age.

The outcome of a clustering analysis critically depends on the number of clusters sought. While data-driven approaches, such as scree plots or silhouette score, can already supply valuable information, in medicine, it is essential to integrate expert knowledge into the interpretation of the results. In our work, we used two clusterings, a coarse clustering with two clusters and a more detailed one with six clusters. Even in the two-cluster approach, there is a distinct separation of two patient groups, showing a relevantly diverging outcome with a more than three-fold increased mortality in one cluster compared to the other. However, a medical interpretation of the two clusters is difficult since clear diagnosis pattern groups did not emerge. This significantly changes when increasing the granularity of the clustering to six clusters. Two clusters (3 and 4) stand out at first sight due to their strongly decreased mortality. In Cluster 3, the predominant procedure was a cardiac catheterization; the patients undergoing this procedure were relatively young, exhibited less concomitant diseases, and did not develop complications to a relevant extent. Cluster 4, in contrast, contains mainly cardiac surgery patients. Examining the description of the MIMIC-III dataset, it stands out that two organizational units are not defined as intensive care units, namely the Coronary Care Unit and the Cardiac Surgery Recovery Unit. It would be conclusive that the patients from these two clusters who showed more or less unremarkable hospital stays were treated on these wards presumably designed for lighter cases. In addition, we note that the above-mentioned cardiac interventions, in the absence of more severe complications, have a very uniform course^28^ so that the “typical courses” are also reflected in the data and therefore facilitate the formation of a cluster. Cluster 5, however, seems to form a homogeneous group of patients suffering from sepsis. This disease affects virtually all organs due to the dysregulated immune response to an infectious agent. Due to that, acute failure of the organs such as kidney, bone marrow (as thrombocytopenia), blood vessels (as septic shock), liver, and dysregulation of electrolytes are much more frequent in this cluster than in the others^29^. Additionally, all possible focuses of infections (lung, urinary tract, intestines, implants) are more prevalent in Cluster 5 than in its complement. Cluster 6, finally, appears to have distinctly unique characteristics, with a significantly prolonged ICU stay and the highest mortality of all clusters. This feature seems to be associated with a need for mechanical ventilation due to various conditions in the vast majority of patients. The common feature of patients in this cluster is not a pulmonary pathology but the requirement for mechanical ventilation itself. Of course, there is a clear predominance of lung-related diagnoses in this cluster, but there are, in the same way, diagnoses that lead to ventilation due to other reasons. For instance, patients with the neurological diagnoses “Intracerebral hemorrhage” and “convulsions”, and patients after cardiac arrest, which can result in severe brain damage, are significantly enriched in this cluster. These neurological causes can lead to impaired consciousness, lack of protective reflexes, or impaired respiratory drive, so it may be necessary to definitively secure the airway and provide sustained ventilation to the patient. But also sepsis-related diagnoses are present in this cluster, whose treatment can be very lengthy. These considerations can explain this cluster’s extended length of stay compared to the remaining ones. Additionally, we observe strong enrichment of procedures typically found in patients requiring long-term medical care, like a temporary tracheostomy, percutaneous endoscopic gastrostomy (PEG), or enteral or parenteral nutrition via infused substances^30,31^. While the four clusters discussed previously can be reasonably interpreted with medical background knowledge, an interpretation of clusters 1 and 2 is more challenging. While clusters 5 and 6, for example, can be identified with a specific keyword, namely “sepsis” and “mechanical ventilation,” no such clear picture emerges in the first two clusters. Looking at the enriched diagnoses, it is eye-catching that most of them have clear sex-specific imbalances^32^, resulting in diagnoses typical for male or female patients heading the list of enriched diagnoses. In contrast, the absolute prevalences of diagnoses are similar to the diagnosis list of the total population and differ only slightly due to sex-related differences in diseases. Thus, it has to be at least considered that clusters 1 and 2 actually form one cluster, which is divided only by the sex of patients, which is part of the input data of the model. The diagnoses contain a common spectrum of cardiovascular diseases, which is barely surprising since nearly half of all U.S. adults have some cardiovascular disease^33^. In the MIMIC-III data set, two of the three top admission diagnoses are cardiovascular diseases, namely “Coronary atherosclerosis” (*414.01*) and “Subendocardial infarction” (*410.71*). Additionally, the performed procedures do not give a clear picture of a specific therapeutic approach for a particular type of patient or disease but represent more or less typical procedures of intensive care medicine. The same applies to the enriched procedures with only placement of a central venous catheter being enriched, which might be due to the lack of this procedure in the less severely affected patients in clusters 3 and 4. We summarize that our method can identify clusters with very distinct features, such as patients that only need to undergo an observation without the need for more invasive procedures or patients that suffer from certain diseases that strongly impact their treatment in a very special way. A very relevant question is the appropriate number of clusters: A higher granularity of clusters might have also identified more specialized patient groups with, e.g., more precise patterns. However, it could also create more artificial clusters that are not usefully interpretable from a medical point of view.

Another interesting result of our work is that the reconstruction quality decreases with the severity of diseases in patients (see Fig. 2). The reasons for this finding still need to be fully understood. A possible explanation for this may be that lab results and vital parameters may be outside of normal ranges for critically ill patients, resulting in fewer training examples for these cases. However, it is also possible that professionals have to correct deregulated physiological functions of these highly sick patients through external interventions, such as increasing their blood pressure by infusing vasopressive agents. Such a nurse-patient feedback control system can never be as precise and smooth as the physiological internal feedback systems. Ultimately, the reason for this pattern in the data needs to undergo further investigation.

The treatment of intensive care patients is a very demanding task for physicians and nurses. In a still widely “analog” process, a vast amount of data points containing information like vital parameters, laboratory test results, blood gas analyses, in addition to results from physical examinations have to be evaluated and classified for their relevance. This mosaic of data and information has to be correctly interpreted to discover critical situations early, allowing a timely treatment. This task can lead to cognitive overload among healthcare workers in intensive care units^34^. Compared to the limited capability of working with multidimensional data of a human brain, a computer-based model is able to handle a multitude of data easily. Dimensionality reduction techniques like autoencoders might be helpful in intensive care settings as well. They could help to recognize minuscule changes in multiple parameters that would frequently escape the healthcare professionals’ attention, but indicate the onset of a severe condition such as sepsis, whose early recognition can be life-saving. The advantage of our method is that, independent of labeling by a medical expert, the structured data already available in electronic health records may be sufficient to detect critical diseases by placing a patient into a certain cluster in the feature space of the autoencoder. To enable such use, experiments regarding the method’s behaviour on incomplete time series would have to be performed.

We welcome more research on unsupervised machine learning in the clinical domain. To improve reproducibility and to facilitate research in this crucial area, we release the code for our method as open source. While supervised methods and tasks are vital tools, unsupervised methods could be more suited for answering open-ended questions, such as the discovery of so far unknown disease patterns. However, it is noteworthy that while the method itself is unsupervised, the method of data collection, namely, everyday clinical practice is not unsupervised. The caregivers’ actions are always part of the data, even if they are not explicitly used as input for a model.

There are many possible avenues for future work: using more of the available dynamic data attributes, an examination of the geometry within the learned feature space, experiments into the relationship between mortality and model performance, or a more in-depth medical and clinical analysis of clusters, especially within a clustering hierarchy. The model and method could also be extended using deep-embedded clustering^35^ or an attention mechanism^27^. Finally, future work could also focus on modifying the proposed method for prognosis or risk stratification.

## Methods

In this section, we present our method for clustering ICU admissions: starting with the input dataset, we then outline the data processing pipeline of our method and show our deep neural network model. Finally, we examine the training scheme and clustering method.

### Data

MIMIC III^36^ is a monocentric database for critical care information, including nurse-verified vital signs, medication administrations, laboratory results, final diagnoses, and other information routinely assessed in ICUs. It contains the medical information of around 60 thousand unique ICU admissions of patients admitted to the Beth Israel Deaconess Medical Center in Boston. The ICU was divided into five subsections focusing on different types of patients: 1. Coronary Care Unit, 2. Cardiac Surgery Recovery Unit, 3. Medical Intensive Care Unit, 4. Surgical Intensive Care Unit, and 5. Trauma Surgical Intensive Care Unit.

Some types of data do not have a timestamp associated with them, those we call static; others are dynamic and may change over time. A patient’s age, sex, or even the diagnoses given by clinicians are static data in MIMIC, while the heart rate, blood pressure, or laboratory results are timestamped and thus dynamic. MIMIC’s public availability has made it a popular dataset for scientific research. This also increases the reproducibility of experiments conducted on the data.

#### Admission selection

Neonates and adults have significantly different medical profiles (e.g. heart rate is much higher in neonates) and receive vastly different care. Thus, neonates and adult ICU patients constitute disparate sets within the population. For this reason, we only include patients aged 22 and up in our study. The age of 22 was chosen because it marks the age at which the FDA classifies patients as adults^37^.

We also exclude admissions that do not have any dynamic data. This results in a total of 49599 admissions for our study (out of the about 60 thousand admissions in MIMIC III). All further statements about the data are based on this selection.

Note that if a single patient has multiple admissions, they are counted as two distinct admissions. This allows our method to examine these admissions in separation (and, more importantly, no admission is discarded).

#### Static data

We include two attributes of static data in the input for our model: the patients’ age in years and their sex. The age is expressed as a floating point number, i.e,. 65.5 means 65 years and 6 months. Our cohort’s median age is 66 years (IQR: 53–78 years). $43.8\%$ of the admitted patients are female. The patient’s sex is expressed as an integer index (0 or 1). Our system is designed to smoothly support other attributes of static data, either numerical (like age) or categorical (like sex).

Except for age and sex, all other static information, like the in-hospital survival of individual admissions is hidden from the model and only used for evaluating the final clusterings.

#### Number of observations

The number of observations (i.e., data points) varies strongly between admissions, with a median of 422 (IQR: 210–849) and a mean of 818 observations. The minimum and maximum number of observations for an admission are 1 and 48583, respectively.

#### Data preprocessing

Since attributes in MIMIC-III are distributed over multiple databases internally, there exist a large number of labeling conflicts, both in the sense that different attributes are given the same label, as well as different labels being given to attributes that refer to the same kind of measurement. In some cases, even single time series are split between attributes, e.g., for ‘Heart Rate,‘ both the attribute ‘220045‘ and the attribute ‘211‘ contain parts of the time series in a single admission. Other attributes, however, are assigned the same label even though they refer to different types of data (e.g., for ‘pH,‘ where attributes are measured in different bodily fluids). We identified 119 such label collisions and resolve them either via fusing the attributes (if they measure the same concept from the same fluid) or split them (if they originate from different fluids or exhibit different data distributions). For details on data preprocessing, as well as a table of label collisions, please refer to our supplementary materials.

#### Data attribute distribution

Not a single attribute of dynamic data is available for every admission. There are 17 attributes that have observations for at least $99\%$ of the admissions: *Hematocrit*, *Creatinine*, *Urea Nitrogen*, *Platelet Count*, *White Blood Cells*, *Hemoglobin*, *MCHC*, *MCH*, *MCV*, *Red Blood Cells*, *RDW*, *Potassium*, *Sodium*, *Chloride*, *Bicarbonate*, *Glucose*, and *Anion Gap* are all results of blood or urine tests. However, the number of distinct attributes quickly increases when lowering the minimum requirement for support: There are 23 attributes at $75\%$, 73 attributes at $50\%$, 244 attributes at $25\%$ and 812 attributes at $1\%$. In total, there are 3106 attributes. This distribution of attribute frequency demonstrates a fundamental problem in clinical data analysis: an overwhelming majority of attributes are only available for a minority of admissions. Due to constraints in compute, we use as input for our model only the 75 attributes with a minimal support of $50\%$. Our method learns to model the temporal characteristics of all of an admission’s time series (up to 75) through a single point in the feature space. In addition to the two static data attributes, this results in 77 data attributes as input to our model.

### Pipeline

**Figure 6:**
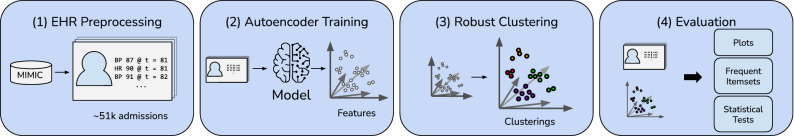
Data processing pipeline. First, medical data is extracted from MIMIC, converted into a format suitable for training the model, and normalized. In the second step, the deep autoencoder model is trained, and a feature vector for each admission is computed. Step 3 is clustering, which first generates clustering candidates through the application of standard clustering algorithms and then ensures the robustness of each clustering using a bootstrapping process. Clusterings that are not robust are filtered out. Finally, the clusterings are evaluated by creating plots, performing statistical tests, and mining frequent ICD codes.

The method consists of four steps: preprocessing, training, clustering, and evaluation, which are summarized in Fig. 6. Preprocessing is performed using scikit-learn^38^ and NumPy^39^ on both static and dynamic data. The available admissions are split into a training set ($90\%)$ and a validation set ($10\%)$ to assess model training and generalization. The split is determined randomly. We scale the input data using quantile transformation^38^, which is more robust to outliers than z-score transformation. First, each data point is mapped to its quantile, resulting in a uniform distribution. Then, the percent-point function (quantile function) is applied, which gives the data a normal distribution with zero mean and unit variance. This is performed for each input feature separately. The normalization is first fit on the training set and then applied to the complete data so that the normalization is not biased concerning the validation set.

In dynamic data, time is represented as the number of minutes that have passed since the beginning of the admission. Inspired by Vaswani et al.^27^, we concatenate onto our dynamic data matrices 64 positional encoding features to express time in addition to a “flat” representation. The positional encodings follow the concept outlined by Vaswani et al. by using sines and cosines alternatingly and with increasing frequencies. This encoding allows the model to learn both relative and absolute times. Since we also keep the normalized time in minutes, this results in 65 temporal features. To test the influence of positional encodings on reconstruction loss, we performed an experiment in which we trained multiple models on 1000 admissions that were randomly sampled for each model. The models using positional encodings achieved, on average, a reconstruction loss of $18\%$ lower than those using a flat time representation.

Since each observation of dynamic data is associated with a point in time in such a way, this representation allows the neural network model to learn how patient conditions may change over time in absolute terms, allowing it to compare, e.g., how much time passes within an hour or within a week.

We interpolate between any two measurements with missing time points in between. The interpolation is performed linearly with regard to the time of measurement. Features without any measurements for an admission are set to the median of the respective attribute. The median is calculated on the train split. Note that we do not re-sample the time series to any grid of fixed step size.

After preprocessing, the model is trained using the training data, and its progress is assessed after each epoch using the validation set. The main product of the training process is a feature space (discussed in more detail in the “Model” section) in which each admission is assigned a feature vector of fixed size, allowing them to be clustered using traditional clustering algorithms within the feature space. Due to randomness in the clustering algorithm, clustering multiple times can result in multiple different clusterings. Each of the produced clusterings is ensured to be robust against minor changes in the input data to improve the robustness of the overall method (see “Clustering” section). Note that the different clusterings are not competing but complement each other to facilitate a more comprehensive understanding of the underlying clinical data. Finally, the clusterings are evaluated, e.g., by analyzing patterns of diagnoses that often co-occur within a cluster.

Diagnoses (in the form of ICD-9 codes) provide insight into the diseases and conditions present in clusters. In order to quantify the notion of diagnoses frequently occurring in clusters, we employ the FP-Max algorithm^40,41^ to mine frequent itemsets of diagnoses. Fisher’s exact test^38,42^ is used to determine if itemsets are positively enriched (i.e., more frequent in the cluster than in the complement), negatively enriched (more frequent in complement), or not enriched. To account for multiple testing, we use Bonferroni correction^43^. The alpha level of $\alpha = 0.01$ is thus divided by the number of tests $n_t = n_{is} \cdot n_c$, where $n_{is}$ is the number of distinct itemsets found, and $n_c$ is the number of clusters within the clustering. Bonferroni correction is performed separately for each clustering.

### Model

Our model follows the design philosophy of autoencoders^44^, which consist of an encoder and a decoder part. Both parts are trained jointly: The encoder learns to compress the high-dimensional input data in such a way that the decoder is able to unpack the compressed representation (the feature space) into the original data again. This compression is lossy, which allows the feature space to capture structure and regularities in the input data. In particular, our model is designed to be able to compress time series of clinical data, the challenge of which lies in producing a feature space without explicit time dependence that yet contains temporal information about the progression of conditions and diseases.

#### Dynamic autoencoder

**Figure 7:**
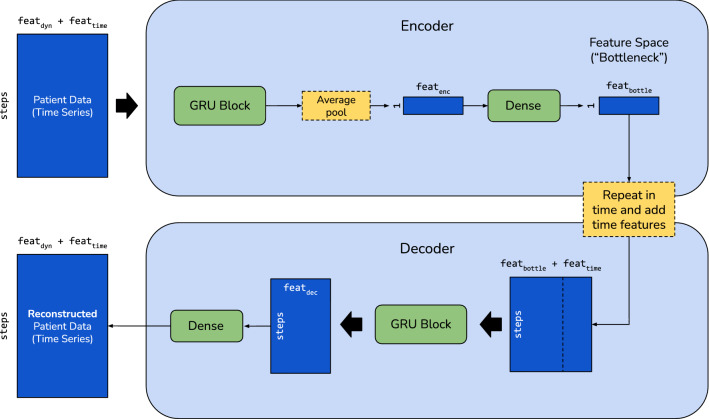
Neural network model. The model consists of an encoder and a decoder part, but these parts are trained jointly. Neural network layers are shown using green boxes with round corners; blue boxes represent tensors, and vector operations like concatenation are shown as yellow boxes with dashed lines. The GRU block is part of the encoder and the decoder and consists of a bidirectional GRU layer, whose outputs are summed for each time step. In the encoder, the resulting features are average pooled over time and then processed using a dense layer, resulting in the encoder’s output: the bottleneck. The decoder repeats the low-dimensional representation in time and concatenates it with the original input’s time features. The same GRU block architecture as for the encoder is used to calculate the temporal dynamics of the time series. Weights are not shared between the two GRU blocks in the model. As a final step, a dense layer is applied to each time step to reconstruct the original features. The model is trained to predict its original input.

As illustrated in Fig. 7, the model comprises two parts: dynamic data encoding and decoding, which are trained jointly.

Dynamic data is presented to the model as a matrix whose rows correspond to time steps and columns to input features. Static data (age, sex) is included in each admission’s matrix as a value that repeats for each time step. Thus, the input to the model is a multivariate time series for each admission.

The most important building block for the model architecture is the GRU^45^ block, consisting of two GRU layers: one reads the input forward and one backward. To improve training stability, we use non-recurrent Dropout regularization^46^ in the GRU layers. The resulting two different temporal features are summed for each time step, and we apply layer normalization^47^. Both the encoder and the decoder use a GRU block, but they do not share weights.

Within the encoder, the features in the GRU block’s output are averaged over time and processed using a dense layer, resulting in a tensor with a single time step called the bottleneck. The bottleneck is named as such because it represents a dimensionally narrow step in the computation. Clustering is later performed based on the contents of the bottleneck, which serves as a feature space.

In the decoder, the bottleneck is repeated in time, so it has the same number of time steps as the input. Then, the original input’s temporal features are appended. This step is necessary because time is not sampled on a regular grid. If the temporal features were not available in the decoder, it would not be able to reconstruct the original time series. A GRU block is then used to uncover temporal behaviors, and a dense layer is applied at each time step in separation. The final output is the reconstructed version of the original time series. The model is trained to make the reconstruction resemble the original multivariate time series as much as possible.

For clustering, the output of the bottleneck is used as a feature space. It carries the information about the admission that the encoder uses to reconstruct the original time series.

### Training

We train our model in Keras^48^ using the Adam optimizer^49^ with a learning rate of 0.00075 and Huber loss with a delta of 1.0. We clip gradient norms to 1 during training. Training is terminated using early stopping with a patience of 8 epochs with respect to the validation loss. We did not observe overfitting: in our final model, the validation loss is slightly lower than the training loss.

Mini batches of size 4 (i.e., four admissions) are assembled by padding shorter admissions to the length of the longest admission in the batch. We employ a masking layer so that our loss and model ignores the padding. Small batch sizes lead to faster convergence for our method.

In addition to the Dropout regularization that is part of the GRU blocks, we add Gaussian noise to the input time series (only the dynamic features, not the temporal features). Note that noise is not added to the prediction target. This functions as a form of data augmentation.

The training of the model with 497588 parameters for 24 epochs takes 1 day and 17 hours on a high-memory CPU machine. After training is complete, we compute the feature space using an encoder variant of the model that shares weights with the trained autoencoder model but ends after the bottleneck layer.

#### Random architecture search

In order to find good values for the model’s hyperparameters (e.g., layer dimensions, RNN type, batch size), it needs to be trained with each combination to determine its performance. However, an exhaustive search would be prohibitively expensive. Both grid and random searches are particularly suited for training in parallel since the hyperparameter configurations do not depend on previous runs. However, random searches have been shown to be more sample-efficient^50,51^ at finding well-performing hyperparameter combinations than grid searches. Our strategy for finding reasonable hyperparameters was to perform random searches on a reduced number of admissions (1000) to gain intuition about the influence of specific hyperparameters. Using this strategy to narrow the search space iteratively allowed us to arrive at the final model presented in this work without performing an exhaustive search over hyperparameter combinations. The final hyperparameters of the model are listed in Table 3.

**Figure 8:**
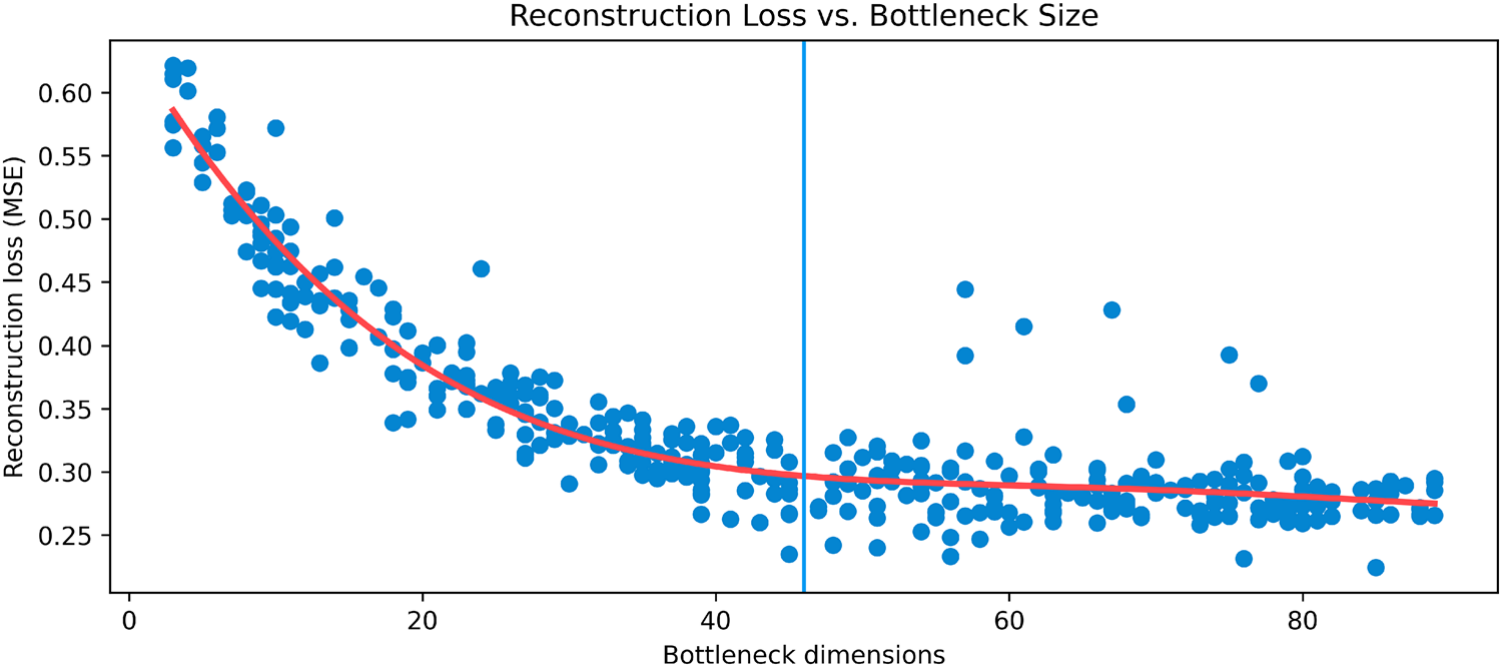
Bottleneck size experiment. Reconstruction loss decreases with increasing size of the bottleneck. However, once a certain threshold of dimensions is reached (at 46 dimensions), the loss stops improving rapidly and only slowly decreases when adding more dimensions to the bottleneck. In red, we show a polynomial of degree 4 that was fit on the data for illustrative purposes. Note that odd bottleneck sizes are rounded up (e.g., 45 becomes 46) to create an even-dimensional feature space for downstream tasks.

The number of dimensions in the bottleneck is of particular interest since it determines the upper limit of information expressed in the feature space. We carried out an experiment to determine the optimal number of bottleneck dimensions (see Fig. 8). We kept all hyperparameters except for the bottleneck size fixed and trained runs with 1000 admissions randomly sampled each time. As a compromise between preserving information (where a higher dimensionality would be preferable) and dimensionality reduction (a lower dimensionality would be preferable), we chose 46 dimensions since this is the smallest number of dimensions that allows reconstruction on par with the best possible reconstruction.

**Table 3:** Model hyperparameter values.

Hyperparameter name	Value
GRU size	158
Bottleneck size	46
Non-recurrent dropout	26.7%
Non-recurrent activation	ELU
Gaussian noise sigma	0.0573

The values are the result of an iterative search process, where ranges of well-performing values are successively made smaller.

### Clustering

Clustering is performed on the 46-dimensional feature space engineered using the autoencoder model. Since the MIMIC dataset is very heterogeneous in terms of demographics, conditions, and diseases, there does not exist a clustering that would reveal every pattern there is in the data. Thus, our goal is to find multiple clusterings that are not competing explanations of the data but complement each other to form a more comprehensive picture. We cluster using the k-medoids algorithm^38^, which has been shown to be more robust to outliers than k-means^52^. For k-medoids, we utilize the k-medoids++ initialization. We examine the clustering results using descriptive statistics of the clusters. These statistics assess the mortality within the clusters, the age distribution, and diagnoses often occurring within a cluster.

**Robustness** To improve the stability of clustering results, we employ the *k-medoids++* initialization method^38,53^. Nonetheless, clustering the same data twice can sometimes lead to entirely different results, and it is then unclear which of the two groupings is to be trusted. To alleviate this problem, we aim to ensure *robust* clusters using a bootstrapping method.

We perform clustering on all admissions for different *k* for k-medoids and then remove trivial clusterings, i.e., those that only contain a single cluster or those where the largest cluster contains more than $90\%$ of admissions. Afterward, we are left with $n_c$ clusters. The same clustering procedure is applied to $n_b = 10$ different *bootstrapped* versions of the feature space, where $70\%$ of the data is randomly sampled without repetition. If we find clusterings similar to the respective clusterings in each bootstrapped version of the data, those clusterings can be considered robust regarding variations in the input data.

We use three metrics for measuring cluster similarity: mutual information, Rand Index, and Jaccard index^38^ denoted with $sim_h$ for $h \in {1, 2, 3}$. Every metric is implemented to be invariant to differences in label literals, e.g., the labeling [0, 0, 1, 1] would be identical to the labeling [1, 1, 0, 0]. We use these metrics to quantify the similarity between each full data clustering $C_{f, i}$ for $i \in [1, n_c]$ and each corresponding bootstrapped clustering $C_{b_j, i}$ for $j \in [1, n_b]$. The metric is only applied to admissions present in each of the bootstrapped clusterings, respectively. For each full clustering $C_{f, i}$ and similarity metric $sim_h$, we calculate a lower bound on the robustness

$$\begin{aligned} rob_{i, h} = P_{10} \left( \left[ sim_{h}(C_{f, i}, C_{b_1, i}), sim_{h}(C_{f, i}, C_{b_2, i}), \dots , sim_{h}(C_{f, i}, C_{b_{n_{b}}, i}) \right] \right) , \end{aligned}$$

where $P_{10}$ denotes the 10th percentile of the similarity values. We also compute a robustness threshold $thresh_{i, h}$, calculated as the 90th percentile of similarities between the real cluster labeling and $n_b$ shuffled instances of it: $sim_h(lab_i, shuffle(lab_i))$. The threshold needs to be calculated for every pair of clustering and similarity metric because the expected similarity when clustering on a bootstrapped version of the data depends on the cluster size distribution and the behavior of the similarity metric. A full data clustering $C_{f, i}$ is considered robust if, for all similarity metrics $sim_h$, it holds that $rob_{i, h} > thresh_{i, h}$.

## Supplementary Information

Supplementary Information.

## Data Availability

The dataset analyzed in this work can be found at https://mimic.physionet.org ^36^. In particular, we analyze the MIMIC-III dataset (https://mimic.mit.edu/docs/iii/).
