# Supplementary material for: Novel architecture for gated recurrent unit autoencoder trained on time series from electronic health records enables detection of ICU patient subgroups
Source: Sci Rep. 2023 Mar 11;13:4053. doi: 10.1038/s41598-023-30986-1 (PMC10008580; doi:10.1038/s41598-023-30986-1)
Supplement: Supplementary file 1 — Supplementary Information. [file 41598_2023_30986_MOESM1_ESM.pdf]

Novel architecture for gated recurrent unit autoencoder trained on time series from electronic health records enables detection of ICU patient subgroups

Kilian Merkelbach[1], Steffen Schaper[2], Christian Diedrich[2], Sebastian Johannes Fritsch[3,4], and Andreas Schuppert[1,*]

[1]JRC-COMBINE, RWTH Aachen University, MTZ, Pauwelsstrasse 19, Level 3, 52074 Aachen, Germany
[2]Pharmacometrics / Modeling & Simulation, Bayer AG - Pharmaceuticals, Leverkusen, Germany
[3]Department of Intensive Care Medicine, University Hospital RWTH Aachen, Pauwelsstrasse 30, 52074 Aachen, Germany
[4]Juelich Supercomputing Centre, Forschungszentrum Juelich, Wilhelm-Johnen-Straße, 52428 Juelich, Germany
[*]schuppert@combine.rwth-aachen.de

Supplementary Information

Data Cleaning

In MIMIC, each column of dynamic data is assigned a label. In order to ensure good data quality, we determined label collisions, i.e., attributes in MIMIC that share the same label. Of the 75 dynamic data attributes present for at least 50% of admissions, 48 did not have a label collision (see Supplementary Table 1). The 27 remaining attributes required either fusing (i.e., joining multiple columns into a single attribute) or splitting (creating multiple attributes from multiple columns with the same label). Fusion was performed by collecting all measurements of the columns to be fused and using mean aggregation where measurements disagreed for a single point in time. According to the MIMIC documentation, we gave preference to the `labevents` table over the `chartevents` table in case of disagreements. Some attributes also exhibit copied values, i.e., one column contains the same measured values as another column but with a short time delay: In this case, we preferred the earlier measurements and discarded the later measurements. In total, 58 raw data columns were used to create 27 dynamic attributes (see Supplementary Table 2).

Label	Column ID
Alanine Aminotransferase (ALT)	50861
Alkaline Phosphatase	50863
Anion Gap	50868
Asparate Aminotransferase (AST)	50878
BUN (6-20)	781
Base Excess	50802
Bicarbonate	50882
Bilirubin, Total	50885
Braden Score	87
Calcium, Total	50893
Calculated Total CO2	50804
Carbon Dioxide	787
Chloride (100-112)	788
Creatine Kinase (CK)	50910
Creatinine (0-1.3)	791
Eye Opening	184
Free Calcium	50808
GCS Total	198
Glucose (70-105)	811
INR(PT)	51237
Lactate	50813
MCH	51248
MCHC	51249
MCV	51250
Magnesium (1.6-2.6)	821
Motor Response	454
NBP Mean	456
NBP [Diastolic]	8441
NBP [Systolic]	455
Neutrophils	51256
Phosphate	50970
Platelets	828
Potassium (3.5-5.3)	829
Potassium, Whole Blood	50822
RDW	51277
Red Blood Cells	51279
Sodium (135-148)	837
SpO2	646
Specific Gravity	51498
Temperature C (calc)	677
Temperature F	678
Urea Nitrogen	51006
Verbal Response	723
WBC (4-11,000)	1127
WBC (4-11,000)	861
White Blood Cells	51301
pCO2	50818
pO2	50821

Supplementary Table 1. Non-colliding dynamic attributes with their labels and column IDs extracted from MIMIC.

Label	Column IDs	Method
Albumin	50862, 227456, 1521	Fused
Arterial Base Excess	224828, 776	Fused
BUN	225624, 1162	Fused
Basophils (Blood)	51146	Split
Chloride	50902, 1523	Fused
Creatinine	50912, 220615, 1525	Fused
Daily Weight	224639, 763	Fused
Eosinophils (Blood)	51200, 3754	Split
Glucose (Blood)	50809, 50931, 1529	Split
Heart Rate	220045, 211	Fused
Hematocrit	51221, 813, 51480	Fused
Hemoglobin	51222, 220228, 50811, 814	Fused
INR	227467, 1530	Fused
Lymphocytes (Blood)	51244	Split
Magnesium	50960, 220635, 1532	Fused
Monocytes (Blood)	51254	Split
PT	51274, 1286	Fused
PTT	51275, 227466, 1533	Fused
Phosphorous	225677, 1534	Fused
Platelet Count	51265, 227457	Fused
Potassium	50971, 1535, 50833	Fused
RBC (Blood)	833	Split
Respiratory Rate	220210, 618	Fused
Sodium	50983, 1536	Fused
WBC (Blood)	220546, 1542	Split
pH (Blood)	50820	Split
pH (Urine)	51491, 51094	Split

Supplementary Table 2. Fused or split attributes. Split attributes are often split based on the bodily fluid the data was collected from, denoted in parentheses within the label. Note that some split attributes are *also* fused, e.g., ‘pH‘ is measured both in the blood (with the single column `50820`) and in urine (with columns `51491` and `51094`, which are fused).

Reconstruction Plots

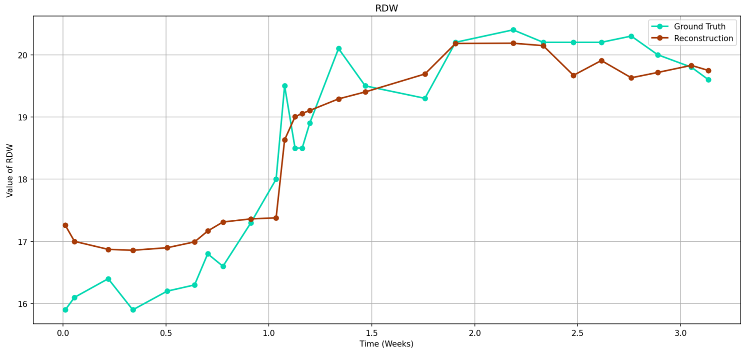

(a) Reconstruction for *RDW*. The MSE of this reconstruction is 0.04. High-frequency information is not reconstructed perfectly, but large movements are captured.

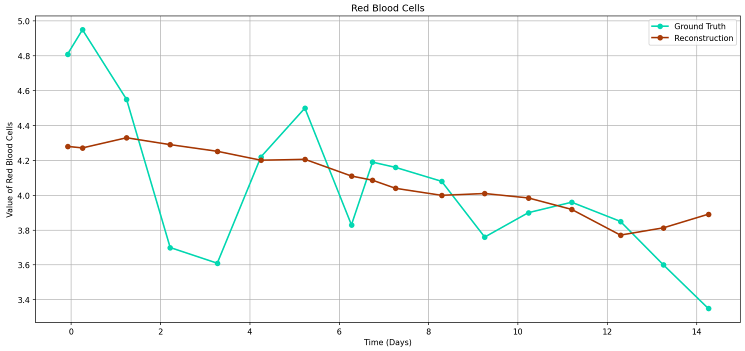

(b) Reconstruction for *Red Blood Cells*. The MSE of this reconstruction is 0.23. This loss is representative of the model, which has a median MSE of 0.2490 over all data.

Supplementary Figure 1. Reconstruction Plots (1). Fidelity of reconstruction using the learned feature space varies between admissions and dynamic data attributes. High-frequency information is often not reconstructed. The plots shown are from different admissions and were chosen to demonstrate different aspects of the model's reconstruction behavior.

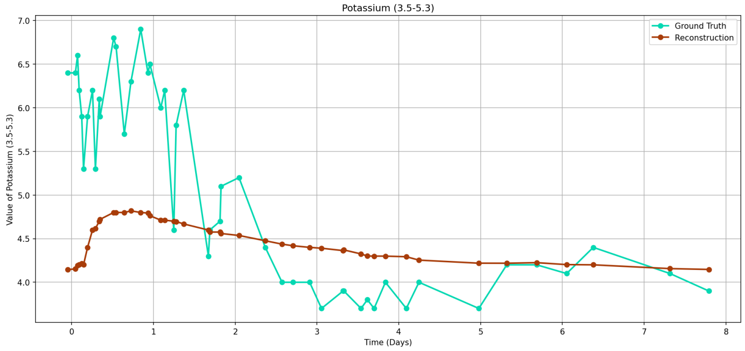

(a) Reconstruction for *Potassium (3.5 - 5.3)*. The MSE of this reconstruction is 1.68. Here, the model did not reconstruct large-scale movements in the ground truth.

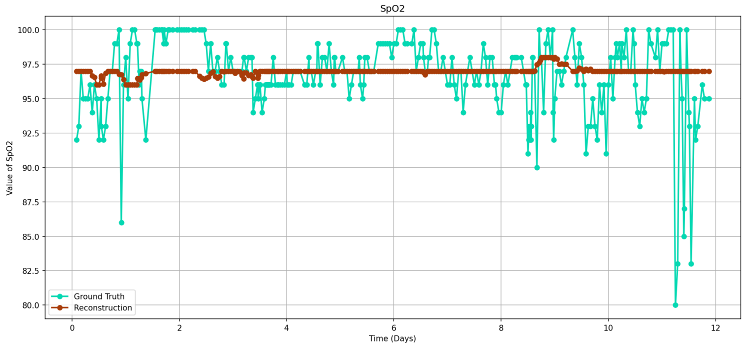

(b) Reconstruction for *SpO2*. The MSE of this reconstruction is 4.5. The reconstruction is almost constant.

Supplementary Figure 2. Reconstruction Plots (2).
